# Safety, Immunogenicity, and Protective Efficacy against Controlled Human Malaria Infection of *Plasmodium falciparum* Sporozoite Vaccine in Tanzanian Adults

**DOI:** 10.4269/ajtmh.17-1014

**Published:** 2018-06-25

**Authors:** Said A. Jongo, Seif A. Shekalaghe, L. W. Preston Church, Adam J. Ruben, Tobias Schindler, Isabelle Zenklusen, Tobias Rutishauser, Julian Rothen, Anneth Tumbo, Catherine Mkindi, Maximillian Mpina, Ali T. Mtoro, Andrew S. Ishizuka, Kamaka Ramadhani Kassim, Florence A. Milando, Munira Qassim, Omar A. Juma, Solomon Mwakasungula, Beatus Simon, Eric R. James, Yonas Abebe, Natasha KC, Sumana Chakravarty, Elizabeth Saverino, Bakari M. Bakari, Peter F. Billingsley, Robert A. Seder, Claudia Daubenberger, B. Kim Lee Sim, Thomas L. Richie, Marcel Tanner, Salim Abdulla, Stephen L. Hoffman

**Affiliations:** 1 Bagamoyo Research and Training Centre, Ifakara Health Institute, Bagamoyo, Tanzania;; 2 Sanaria Inc., Rockville, Maryland;; 3 Swiss Tropical and Public Health Institute (Swiss TPH), Basel, Switzerland;; 4 University of Basel, Basel, Switzerland;; 5 Vaccine Research Center (VRC), National Institute of Allergy and Infectious Diseases, National Institutes of Health, Bethesda, Maryland;; 6 Protein Potential LLC, Rockville, Maryland

## Abstract

We are using controlled human malaria infection (CHMI) by direct venous inoculation (DVI) of cryopreserved, infectious *Plasmodium falciparum* (Pf) sporozoites (SPZ) (PfSPZ Challenge) to try to reduce time and costs of developing PfSPZ Vaccine to prevent malaria in Africa. Immunization with five doses at 0, 4, 8, 12, and 20 weeks of 2.7 × 10^5^ PfSPZ of PfSPZ Vaccine gave 65% vaccine efficacy (VE) at 24 weeks against mosquito bite CHMI in U.S. adults and 52% (time to event) or 29% (proportional) VE over 24 weeks against naturally transmitted Pf in Malian adults. We assessed the identical regimen in Tanzanians for VE against PfSPZ Challenge. Twenty- to thirty-year-old men were randomized to receive five doses normal saline or PfSPZ Vaccine in a double-blind trial. Vaccine efficacy was assessed 3 and 24 weeks later. Adverse events were similar in vaccinees and controls. Antibody responses to Pf circumsporozoite protein were significantly lower than in malaria-naïve Americans, but significantly higher than in Malians. All 18 controls developed Pf parasitemia after CHMI. Four of 20 (20%) vaccinees remained uninfected after 3 week CHMI (*P* = 0.015 by time to event, *P* = 0.543 by proportional analysis) and all four (100%) were uninfected after repeat 24 week CHMI (*P* = 0.005 by proportional, *P* = 0.004 by time to event analysis). *Plasmodium falciparum* SPZ Vaccine was safe, well tolerated, and induced durable VE in four subjects. Controlled human malaria infection by DVI of PfSPZ Challenge appeared more stringent over 24 weeks than mosquito bite CHMI in United States or natural exposure in Malian adults, thereby providing a rigorous test of VE in Africa.

## INTRODUCTION

In 2015 and in 2016, there were an estimated 429,000–730,500 deaths caused by malaria.^1–3^ *Plasmodium falciparum* (Pf) is the cause of > 98% of malaria deaths and > 80% of malaria cases in sub-Saharan Africa. Our goal is to field a vaccine that will prevent infection with Pf and thereby prevent all manifestations of Pf malaria and parasite transmission from humans to mosquitoes.^4^

*Plasmodium falciparum* sporozoites (SPZ) are the only immunogens that have ever prevented Pf infection in > 90% of subjects.^5–7^ Sanaria^®^ PfSPZ Vaccine (Sanaria Inc., Rockville, MD) is composed of radiation-attenuated, aseptic, purified, cryopreserved PfSPZ.^8,9^ When administered by rapid intravenous injection, PfSPZ Vaccine protected 100% (6/6) of malaria-naïve subjects in the United States against mosquito bite–controlled human malaria infection (CHMI) with Pf parasites similar to those in the vaccine (homologous) 3 weeks after the last immunization,^10^ and 65% at 24 weeks.^11^ Protection was durable against homologous mosquito bite CHMI for at least 59 weeks^12^ and heterologous (parasites different than in vaccine) mosquito bite CHMI for at least 33 weeks.^13^ PfSPZ Vaccine also prevented naturally transmitted heterogeneous Pf in adults in Mali for at least 24 weeks (vaccine efficacy [VE] 52% by time to event and 29% by proportional analysis).^14^

We used the same dosage regimen as in the United States and Mali to evaluate the tolerability, safety, immunogenicity, and VE of PfSPZ Vaccine in young adult male Tanzanians. Previously, we had conducted the first modern CHMI in Africa and showed that injection of aseptic, purified, cryopreserved PfSPZ, Sanaria^®^ PfSPZ Challenge, consistently infected Tanzanian volunteers and subsequently repeated in multiple other countries.^15–21^ In this study, we took advantage of this capability to assess VE of PfSPZ Vaccine by CHMI with PfSPZ Challenge (NF54). The same PfSPZ Vaccine dosage regimen was less immunogenic and protective in Tanzanians than in Americans,^11^ and VE against homologous CHMI in Tanzania was lower (or similar) to VE against intense field exposure to heterogeneous Pf parasites in Mali.^14^

## MATERIAL AND METHODS

### Study design and population.

This double-blind, randomized, controlled trial was conducted in Bagamoyo, Tanzania, between April 2014 and August 2015. Sixty-seven healthy male volunteers of 18–35 years of age were recruited from higher learning institutions in Dar es Salaam. After initial screening, prospective volunteers were invited to the Bagamoyo Clinical Trial Unit of the Ifakara Health Institute (IHI) to complete informed consent and screening.

All had to complete a 20-question assessment of trial understanding with a 100% correct response rate on the first or second attempt (Supplemental Table 1) to be eligible. Volunteers were screened using predetermined inclusion and exclusion criteria (Supplemental Tables 2 and 3). History of malaria in the previous 5 years or antibodies to Pf exported protein 1 (PfEXP1) by an enzyme-linked immunosorbent assay (ELISA) above a level associated with a single, recent Pf infection by CHMI^19^ (see the Antibody assays section) were the exclusion criteria. Hematology, biochemistry, and parasitology testing, including malaria thick blood smear (TBS), stool, and urine by microscopy was carried out. Tests for human immunodeficiency virus and hepatitis B and C were performed after counseling; volunteers were excluded if positive and referred for evaluation and management by appropriate local physicians. Volunteers were excluded if they had significant abnormalities on electrocardiograms.

The trial was performed in accordance with Good Clinical Practices. The protocol was approved by institutional review boards (IRBs) of the IHI (Ref. No. IHI/IRB/No:02-2014), the National Institute for Medical Research Tanzania (NIMR/HQ/R.8a/Vol.IX/1691), the Ethikkommission Nordwest-und Zentralschweiz, Basel, Switzerland (reference number 261/13), and by the Tanzania Food and Drug Authority (Ref. No. TFDA 13/CTR/0003); registered at Clinical Trials.gov (NCT02132299); and conducted under U.S. FDA IND application.

### Investigational products (IPs).

The IPs were Sanaria^®^ PfSPZ Vaccine^8–14^ and Sanaria^®^ PfSPZ Challenge.^15–20^ PfSPZ Vaccine consists of aseptic, purified, vialed, metabolically active, nonreplicating (radiation attenuated), cryopreserved PfSPZ (NF54 strain). It was stored, thawed, diluted, and administered by direct venous inoculation (DVI) in 0.5 mL through a 25-gauge needle.^11,14,18,20^ PfSPZ Challenge is identical to PfSPZ Vaccine except it is not radiation attenuated. It was handled and administered like PfSPZ Vaccine. Preparation of IPs was supervised by the study pharmacist. After labeling the syringe, the pharmacist handed it to the clinical team through a window.

### Allocation and randomization.

**Figure 1.**
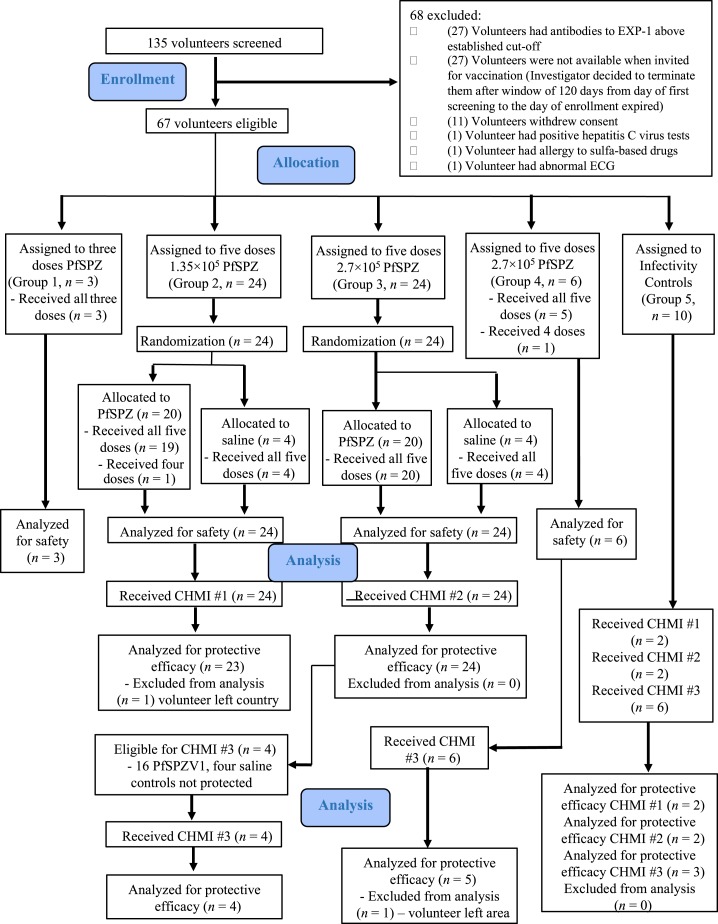
Volunteer participation (CONSORT 2010 diagram). This figure appears in color at www.ajtmh.org.

Volunteers were allocated to five groups (Table 1; Figure 1). Forty-nine received PfSPZ Vaccine and eight normal saline (NS). Ten were additional infectivity controls. The clinical team and volunteers were blinded to assignment to vaccine or NS until study end.

**Table 1:** Demographic characteristics of volunteers

	Vaccinees	Normal saline controls	Infectivity controls
Number of volunteers	49	8	10
Percentage males	100%	100%	100%
Mean age in years (range)	24 (20, 30)	23 (20, 28)	25 (21, 28)
Percentage Africans	100%	100%	100%
Mean body mass index (range)	22.33 (18.00, 29.70)	21.91 (19.00, 24.20)	21.68 (18.40, 24.30)
Number (%) heterozygous for alpha thalassemia	22 (44.9%)	4 (50%)	5 (50%)
Number (%) with LTBI* (QuantiFERON positive)	17 (34.7%)	3 (36.5%)	1 (10%)
Number (%) positive on screening of urine or stool for parasitic infection	0 (0%)	1 (12.5%)	0 (0%)
Number (%) students	49 (100%)	8 (100%)	10 (100%)

* Latent tuberculosis infection.

#### Group 1.

Three volunteers received consecutive doses of 3 × 10^4^, 1.35 × 10^5^, and 2.7 × 10^5^ PfSPZ of PfSPZ Vaccine at 4-week intervals to assess safety (Group 1).

#### Groups 2 and 3.

Volunteers were randomized to receive 1.35 × 10^5^ PfSPZ of PfSPZ Vaccine (*N* = 20) or NS (*N* = 4) (Group 2), or 2.7 × 10^5^ PfSPZ of PfSPZ Vaccine (*N* = 20) or NS (*N* = 4) (Group 3) at 0, 4, 8, 12, and 20 weeks.

#### Group 4.

Six volunteers were immunized with 2.7 × 10^5^ PfSPZ of PfSPZ Vaccine on the same schedule as Group 3.

#### Group 5.

Ten volunteers served as unblinded infectivity controls during CHMIs (see in the following paragraph): two with CHMI #1, two with CHMI #2, and six with CHMI #3.

### Vaccine efficacy.

#### Controlled human malaria infection.

Vaccine efficacy was assessed by CHMI by DVI of 3.2 × 10^3^ PfSPZ of PfSPZ Challenge. Controlled human malaria infection #1 was 3 weeks after the last immunization in Group 2. Controlled human malaria infection #2 was 3 weeks after the last immunization in Group 3. Controlled human malaria infection #3 was 24 weeks after the last immunization in Groups 3 and 4 and included the four volunteers in Group 3 who did not develop parasitemia after CHMI #2 and the six Group 4 volunteers. Volunteers were inpatients from day 9 after PfSPZ Challenge injection for observation until diagnosed and treated for malaria or until day 21; daily outpatient monitoring for TBS-negative volunteers continued until day 28. Thick blood smears were obtained every 12 hours on days 9–14 after CHMI and daily on days 15–21 until positive or until day 21. Thick blood smears could be performed more frequently, if volunteers had symptoms/signs consistent with malaria. After initiation of treatment, TBSs were assessed until two consecutive daily TBSs were negative and on day 28.

#### Detection of Pf parasites and parasite DNA.

Slide preparation and reading for TBSs were performed as described.^19^ Sensitivity was 2 parasites/μL blood unless the volunteer was symptomatic, in which case four times as many fields were read. Parasitemia was also determined by quantitative polymerase chain reaction (qPCR) with sensitivity of 0.1 parasites/μL blood based on a multiplex assay detecting *Plasmodium* spp. 18S genes and the human RNaseP gene as endogenous control.^22^ A second, more sensitive qPCR assay with a sensitivity of 0.05 parasites/μL blood and targeting the Pf-specific telomere-associated repetitive element 2^23^ was used to reanalyze all samples that were negative by 18S-based qPCR. After the start of CHMI, the time of first blood sample positivity by qPCR was used to determine infection status and for the calculation of prepatent period. Volunteers were continuously monitored by qPCR until malaria treatment based on TBS positivity. The World Health Organization International Standard for Pf DNA Nucleic Acid Amplification Techniques (NIBSC, Hertfordshire, United Kingdom) was used as standard for calculation of parasite densities. DNA was extracted from 100 μL whole blood and eluted with 50 μL Elution Buffer using Quick-gDNA Blood MicroPrep Kit (Zymo Research, Irvine, CA). Blood samples were analyzed retrospectively by qPCR after storing at −80°C after the conclusion of CHMIs. To exclude field strain infections, parasite genotyping was performed on samples randomly chosen as described.^24^ In all cases in which TBS was negative and qPCR was considered positive, two consecutive samples were positive by qPCR.

### Adverse events (AEs).

Volunteers were observed as inpatients for 48 hours after administration of IP and discharged with diaries and thermometers for recording AEs and temperatures and followed with daily telephone calls. Symptoms and signs (solicited and unsolicited) were recorded and graded by physicians: mild (easily tolerated), moderate (interfere with normal activity), severe (prevents normal activity), or life threatening. Axillary temperature was grade 1 (> 37.5–38.0°C), grade 2 (> 38.0–39.0°C), grade 3 (> 39.0–40.0°C), or grade 4 (> 40.0°C). Hematological and biochemical abnormalities were also assessed using standard clinical assays.

During the first 7 days after injection of IPs, prespecified local (site of injection) and systemic AEs were solicited. Open-ended questioning was used to identify unsolicited AEs through day 28 (Supplemental Table 4). All AEs were assessed for severity and relatedness to IP administration. Adverse events were classified as definitely related, probably related, possibly related, unlikely to be related, and not related. Definitely, probably, and possibly were considered to be related. Unlikely to be related and not related were considered to be unrelated. For CHMIs, volunteers returned on day 9 for admission to the ward for diagnosis and treatment of malaria. Events during the 8–28 day period were assessed for relationship to Pf infection and considered related if the event was within 3 days before and 7 days after TBS was first positive.

### Antibody assays.

Sera were assessed for antibodies by ELISA, immunofluorescence assay (aIFA), and inhibition of sporozoite invasion (ISI) assay as described (see Supplemental Table 5).^25^ For ELISAs, the results are reported as the serum dilution at which the optical density (OD) was 1.0. Enzyme-linked immunosorbent assay for PfEXP1 was used to screen volunteers for possible malaria exposure (Supplemental Table 6). Any subject with an OD 1.0 of ≥ 600 was excluded. This was because we had previously determined in Tanzanians who underwent CHMI^19^ that antibodies to PfEXP1 at this level were a sensitive indicator of recent Pf infection (unpublished).

### T-cell assays.

T-cell responses in cryopreserved peripheral blood mononuclear cells (PBMC) were measured by flow cytometry in a single batch after the study as described.^12^ After stimulation, cells were stained as described.^26^ The staining panels are in Supplemental Table 7 and antibody clones and manufacturers are in Supplemental Table 8. All antigen-specific frequencies are reported after background subtraction of identical gates from the same sample incubated with control antigen. Data were analyzed with FlowJo v9.9.3 (TreeStar, Ashland, OR) and graphed in Prism v7.0a (GraphPad, San Diego, CA).

### Statistical analysis.

Comparisons of categorical variables between groups were analyzed using 2-tailed Fisher’s exact test. Comparisons of continuous variables between groups were analyzed by 2-tailed nonparametric tests. For multiple group comparisons, the Kruskal–Wallis test was used. Time to event was assessed by the Kaplan–Meier curves and log-rank test. Vaccine efficacy by time to event was quantified using Cox proportional hazards ratios. Time to event data were analyzed from CHMI injection until positive TBS result or positive qPCR result. Controlled human malaria infection follow-up period lasted until day 28 after CHMI injection. Analyses of immunological data are described with the data.

### Role of the funding source.

The funders were involved in study design, study management, data collection, data analysis, data interpretation, and writing the report. Salim Abdulla and Stephen L. Hoffman had full access to all data in the study and final responsibility for decision to submit for publication.

## RESULTS

### Study population and experience with DVI.

Fifty-seven Tanzanian men (Table 1; Figure 1) met the criteria (Supplemental Tables 2 and 3) and received PfSPZ Vaccine (*N* = 49) or NS (*N* = 8). All volunteers had AA hemoglobin and normal G6PD activity. Thirty-one volunteers (46%) were heterozygous for α-thalassemia; 21 had evidence of latent tuberculosis infection by Quantiferon testing, but showed no evidence of active tuberculosis. One volunteer (group 2, NS) had *Strongyloides stercoralis* on screening and was successfully treated before vaccination (Table 1).

Of 237 immunizations with PfSPZ Vaccine, 234 were completed with a single injection (98.7%). Two hundred and thirty injections (97.0%) were considered painless by the volunteer. For NS subjects, 39 of 40 immunizations (97.5%) were completed in a single injection and 39 of 40 (97.5%) considered painless by the volunteer. The nurse performing immunizations considered the procedure to be simple in 265 of 273 single injections (97.1%).

One subject in Group 2 received four immunizations. The third immunization was withheld while the subject was evaluated for what was diagnosed as benign ethnic neutropenia.^27,28^ One subject in Group 4 missed his second immunization when he left town. All other subjects (other than Group 1 and added infectivity controls) received five immunizations.

### Safety.

Among 49 volunteers who received 237 doses of PfSPZ Vaccine, there were 17 solicited AEs possibly related to IP (17/237 = 7.2%) in 10 of the 49 vaccinees (20.4%) (Table 2). Among eight volunteers who received 40 doses of NS, there were two solicited AEs possibly related to IP (2/40 = 5.0%) in one of the eight controls (12.5%) (Table 2). There were no AEs considered by the clinicians to be probably or definitely related to IP. There were no local or serious AEs. One episode each of headache and fever were grade 2; all other solicited AEs were grade 1. None of the comparisons of AEs between vaccinees and controls or between Group 2 (1.35 × 10^5^ PfSPZ) and Groups 3 and 4 (2.7 × 10^5^ PfSPZ) showed statistically significant differences (Table 2). Twenty-six of 49 vaccinees (53.1%) experienced 43 unsolicited AEs (0.88/individual) in the 28 days following injections #1–#4 and the 21 days before CHMI after injection #5. Seven of eight controls (87.5%) experienced 14 unsolicited AEs (2/individual) during this period. None of these unsolicited AEs recorded within 28 days of an immunization was considered related to IP.

**Table 2:** Solicited AEs by group considered possibly* related to administration of the investigational product during the first 7 days post immunization

	Group 1 (dose escalation)	Group 2 (1.35 × 10^5^ PfSPZ)	Group 3 (2.7 × 10^5^ PfSPZ)	Group 4 (2.7 × 10^5^ PfSPZ)	Total PfSPZ vaccine	NS controls
Number of volunteers	3	20	20	6	49	8
Total number of injections	9	99	100	29	237	40
Number of local AEs	0	0	0	0	0	0
Numbers of systemic AEs (% of total immunizations)						
All	1 (11%)	10 (10.1%)	6 (6%)	0	17 (7.2%)	2 (5.0%)
Headache*	1 (11%)	7 (7%)†	2 (2%)	0	10 (4.2%)	1 (2.5%)
Abdominal pain	0	2 (2%)	1 (1%)	0	3 (1.3%)	0
Chills	0	0	1 (1%)	0	1 (0.4%)	0
Fever	0	0	2 (2%)	0	2 (0.8%)	0
Diarrhea	0	0	0	0	0	1 (2.5%)
Chest pain	0	1 (1%)	0	0	1 (0.4%)	0
Other	0	0	0	0	0	0
Systemic AEs - no. volunteers with ≥ 1 event (% of volunteers)						
Any	1 (33%)	7 (35%)	2 (10%)	0	10 (20.4%)	1 (13%)
Headache	1 (33%)	6 (30%)	2 (10%)	0	9 (18.4%)	1 (13%)
Abdominal pain	0	2 (10%)	1 (5%)	0	3 (6.1%)	0
Chills	0	0	1 (5%)	0	1 (2.0%)	0
Fever	0	0	2 (10%)	0	2 (4.1%)	0
Diarrhea	0	0	0	0	0	1 (13%)
Chest pain	0	1 (5%)	0	0	1 (2.0%)	0
All other	0	0	0	0	0	0

AEs = adverse events; PfSPZ = *Plasmodium falciparum* sporozoites. There were no significant differences between vaccinees as compared with normal saline (NS) controls for any or all AEs. All AEs were grade 1, except one headache and one fever. Local solicited AEs: injection site pain, tenderness, erythema, swelling, or induration. Systemic solicited AEs: allergic reaction (rash, pruritus, wheezing, shortness of breath, bronchospasm, allergy-related edema/angioedema, hypotension, and anaphylaxis), abdominal pain, arthralgia, chest pain/discomfort, chills, diarrhea, fatigue, fever, headache, malaise, myalgia, nausea, pain (other), palpitations, shortness of breath, and vomiting.

* All AEs were considered possibly related. None were considered probably or definitely related.

† 4/7 episodes of headache occurred after the third vaccine dose and did not recur with fourth or fifth doses. No factor was identified to account for this apparent clustering of headache.

Laboratory abnormalities occurred at roughly equal rates comparing PfSPZ Vaccine recipients and controls, except for leukocytosis and eosinophilia, which were more frequent in controls (Table 3). There was no apparent explanation for these differences. A cyclic variation in total bilirubin following each immunization was observed equally in volunteers receiving vaccine or NS that was attributed to enriched diet, as the volunteers were transported to Bagamoyo from Dar es Salaam during the periods of immunization and CHMI and were amply fed (see Supplemental Figure 1). In Dar es Salaam, malaria transmission is low. No volunteer had malaria during screening or during the trial other than from CHMI.

**Table 3:** Summary of abnormal laboratory values and severity grades

	Vaccinees in Group 2 (1.35 × 10^5^ PfSPZ) (*N* = 20)		Vaccinees in groups 3 and 4 (2.7 × 10^5^ PfSPZ) (*N* = 26)		NS controls (*N* = 8)		*P* values: vaccinees (*N* = 46) vs. controls (*N* = 8)
Laboratory parameter	No.	%	No.	%	No.	%	
Leukocytosis	1	5	2	7.7	3	37.5	0.0358
Leukopenia	6	30	7	27	1	12.5	> 0.05
Neutropenia	6	30	5	19	2	25	> 0.05
Lymphopenia	3	15	3	11.5	2	25	> 0.05
Eosinophilia	0	0	2	7.7	3	37.5	0.0194
Decreased hemoglobin	1	5	0	0	0	0	> 0.05
Thrombocytopenia	1	5	0	0	0	0	> 0.05
Elevated creatinine	2	10	4	15.4	2	25	> 0.05
Low total bilirubin	4	20	7	27	1	12.5	> 0.05
Elevated total bilirubin	2	10	2	7.7	2	25	> 0.05
Elevated alkaline phosphatase	1	5	2	7.7	0	0	> 0.05
Elevated alanine aminotransferase	3	15	5	19	2	25	> 0.05
Elevated aspartate aminotransferase	0	0	3	11.5	0	0	> 0.05

PfSPZ = *Plasmodium falciparum* sporozoites. *P* values calculated using Fisher’s exact test (2-tailed). One volunteer who received saline developed Grade 3 eosinophilia attributed to *Strongyloides stercoralis* infection, which improved with anthelminthic therapy. This volunteer had a baseline of mild eosinophilia, which persisted throughout the clinical trial. All other laboratory abnormalities were Grade 2 or less. There was no association between laboratory abnormalities and time after a dose or increasing number of doses. Three abnormalities during immunization were deemed clinically significant or Grade 3. One was diagnosed as benign ethnic neutropenia, one was lymphopenia associated with an infected foot laceration, and one was eosinophilia associated with *Fasciolopsis buski* and *S. stercoralis* infection. Lymphopenia and eosinophilia resolved with treatment. Two Group 4 volunteers had asymptomatic hookworm infections diagnosed before controlled human malaria infection; one was coinfected with *Enterobius vermicularis*.

### Tolerability, safety, and VE during CHMI.

Forty-six vaccinees, eight NS controls, and 10 added infectivity controls underwent homologous CHMI. All subjects were negative by TBS and qPCR for Pf infection on the day of CHMI. Two volunteers were excluded from primary analysis—a Group 2 volunteer who left the area 2 days after administration of PfSPZ Challenge and a Group 4 volunteer who left 9 days after. Both volunteers were located and treated preemptively.

#### Tolerability and safety of administration of PfSPZ challenge.

Controlled human malaria infection was well tolerated with no local solicited AEs and three systemic solicited AEs (grade 1 headache in Group 3, grade 2 headache in Group 4, and grade 1 arthralgia in an infectivity control) in the 7 days post-administration of PfSPZ Challenge.

#### Parasitemia.

##### Controls.

**Figure 2.**
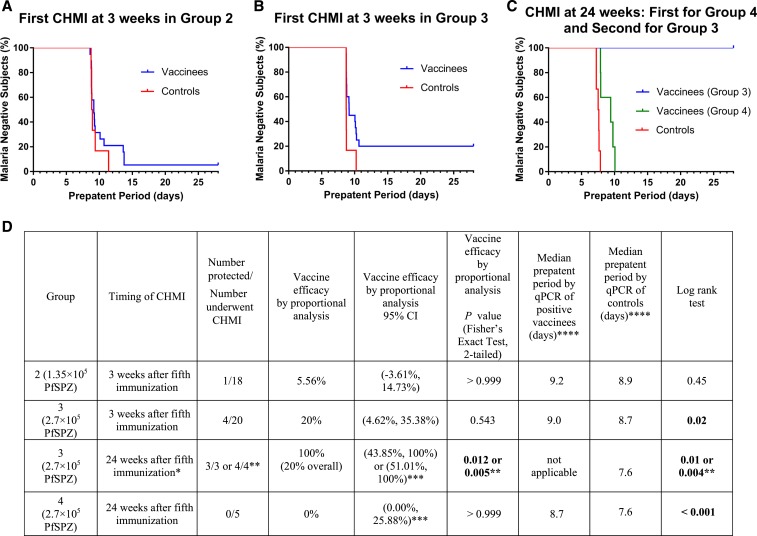
Kaplan–Meier survival curves in immunized volunteers vs. controls as assessed by quantitative polymerase chain reaction (qPCR). Kaplan–Meier curves in volunteers undergoing controlled human malaria infection (CHMI) 3 weeks after the last of five doses with 1.35 × 10^5^ (Group 2) (**A**) or 2.7 × 10^5^ (Group 3) (**B**) *Plasmodium falciparum* Sporozoites (PfSPZ) of PfSPZ Vaccine. Panel (**C**) volunteers undergoing either first (Group 4) or second (Group 3) CHMI 24 weeks after the fifth immunization with 2.7 × 10^5^ PfSPZ of PfSPZ Vaccine. (**D**) Vaccine efficacy and prepatent period results. *This was the second CHMI for the 4 volunteers in Group 3 who were protected after the first CHMI at 3 weeks. **One volunteer was inappropriately treated on day 13 for a false positive TBS. Without this volunteer, 3/3 protected. With this volunteer 4/4 were protected. ***Confidence intervals were calculated using Wilson’s score interval. ****Volunteers in CHMI #1 and #2 (3 week CHMI in Groups 2 and 3) had specimens first acquired on day9. Volunteers in CHMI #3 (24 week CHMI in Groups 3 and 4) had specimens first acquired on day 8. This figure appears in color at www.ajtmh.org.

The 18 NS and infectivity controls developed Pf infection after CHMI (16 TBS and qPCR positive and two TBS negative and qPCR positive) (Figure 2A–D and Supplemental Table 9). These included four NS and two infectivity controls in CHMI #1, the same in CHMI #2, and six infectivity controls in CHMI #3. All received the same lot of PfSPZ Challenge. One isolate of those positive from CHMI #1, one from CHMI #2, and four from CHMI #3 were genotyped,^24^ and all parasites tested were PfNF54. Vaccine efficacy was calculated based on the results of qPCR assays from the six controls in CHMI #1, CHMI #2, and CHMI #3 individually (Figure 2D).

##### Group 2 (1.35 × 10^5^ PfSPZ).

Seventeen of 18 volunteers who received five doses and 1/1 volunteer who received four doses developed parasitemia (Figure 2A), 15 positive by TBS and qPCR, and 3 by qPCR only (CHMI #1) (Supplemental Table 10). One volunteer was negative through day 28 by TBS and qPCR. Vaccine efficacy by proportional analysis was 5.56% (95% confidence interval [CI]: 3.61%, 14.73%; *P* > 0.99, Fisher’s exact test, 2-tailed). There was no significant delay in parasitemia by qPCR in the vaccinees as compared with controls (*P* = 0.4481 by log rank).

##### Group 3 (2.7 × 10^5^ PfSPZ).

First CHMI at 3 weeks (CHMI #2): 16/20 volunteers who received five doses developed parasitemia (Figure 2B), all positive by TBS and qPCR; four volunteers were negative through day 28 by TBS and qPCR. Vaccine efficacy by proportional analysis was 20% (95% CI: 4.62%, 35.38%; *P* = 0.543). There was a delay in the onset of parasitemia in vaccinees as compared with controls (*P* = 0.015 by log rank).

Second CHMI at 24 weeks (CHMI #3): The four uninfected volunteers from the first CHMI underwent a second CHMI 24 weeks after the last vaccine dose (Figure 2C). Three were negative by TBS and qPCR through day 28 day. The fourth volunteer, who was asymptomatic, was reported to have a positive TBS on day 12 and treated. The sample with positive TBS was negative by retrospective qPCR. Reevaluation of the TBS indicated an error in slide reading (false-positive). Vaccine efficacy by proportional analysis at this time point was 100% (for 3/3 and 4/4 protected: 95% CI: 43.8%, 100%, and 51.01%, 100%; *P* = 0.012 and 0.005, respectively). However, given the 20% VE at 3 weeks by proportional analysis, overall VE by proportional analysis was considered to be 20%.

##### Group 4 (2.7 × 10^5^ PfSPZ).

First CHMI at 24 weeks after the last vaccine dose (CHMI #3): 4/5 vaccinees developed parasitemia by TBS and qPCR. The fifth was negative by TBS, but positive by qPCR (see Supplemental Table 10). There was one excluded volunteer (see the previous paragraph). Vaccine efficacy by proportional analysis was 0% (*P* > 0.99%). There was a significant delay in the onset of parasitemia by qPCR in vaccinees as compared with controls (*P* = 0.001 by log rank).

#### α-thalassemia.

Volunteers heterozygous for α-thalassemia were no more likely to be TBS negative and qPCR positive than volunteers without α-thalassemia (three of 27 versus three of 34, *P* = 1.0). Protection from CHMI did not correlate with α-thalassemia status; 3/37 with normal hemoglobin and 2/29 heterozygous for α-thalassemia were protected.

#### Prepatent periods and parasite densities.

Although the median prepatent periods by TBS in controls in each CHMI group (12.5, 13.0, and 12.0, respectively) were shorter than in the vaccinees in Groups 2–4 (14.0, 14.0, and 15.3 days, respectively), these differences did not reach the level of statistical significance (*P* = 0.486, *P* = 0.491, and *P* = 0.333, respectively) (Supplemental Table 9). The prepatent periods by qPCR in vaccineees in Group 3 (3 and 24 week CHMIs) and Group 4 (24 week CHMI) were significantly longer than in the respective controls (Figure 2D). The parasite densities by qPCR and TBS at the time of diagnosis for each individual are in Supplemental Table 10. The median parasite density in controls versus vaccinees at the time of first positivity were 0.5 versus 0.4 parasites/μL for qPCR (*P* = 0.5714) and 11.2 versus 15.0 parasites/μL for TBS (*P* = 0.1492).

#### Tolerability and safety of parasitemia during CHMI.

##### Controls.

Sixteen controls developed parasitemia by TBS; 9 (56%) never had symptoms (Supplemental Table 11). Headache occurred in 7/7 symptomatic individuals. One of two control volunteers only positive by qPCR did not have any symptoms; the second had headache 8 days after qPCR spontaneously reverted to negative. No volunteer had symptoms at the time of first positive qPCR.

##### Vaccinees.

Thirty-five immunized volunteers developed parasitemia by TBS; 20 (57%) never had symptoms. Three volunteers had temperature > 39.0°C; all other clinical manifestations were grade 1 or 2. Fever (28.6%) and headache (31.4%) were most common. Compared with controls, elevated temperature was more common in vaccinees with positive TBSs (9/35 versus 0/16, *P* = 0.043). There was no significant difference in the frequency of headache between controls and vaccinees. In the three volunteers in Group 2 who were qPCR positive and TBS negative, one developed headache 3 days after qPCR positivity. No volunteer had symptoms at the time of first positive qPCR.

##### Clinical laboratories.

No unexpected changes were observed following CHMI. Declines in lymphocyte counts were observed in TBS positive controls and vaccinees (mean decline 1,110 ± 720 cells/μL and 1,180 ± 680 cells/μL, respectively) on day of first positive TBS. Absolute lymphocyte counts less than 1,000 cells/μL were observed in 8/16 and 16/35 TBS positive controls and vaccinees. All lymphocyte counts returned to the baseline by day 28. There were mild decreases in platelet counts in TBS positive subjects, but all platelet counts were > 100 × 10^3^ cells/μL.

#### Treatment.

Volunteers with positive TBSs were treated with either atovaquone/proguanil (*N* = 43) or artemether/lumefantrine (*N* = 8) within 24 hours of first positive TBS. Normal saline and infectivity controls who were TBS negative (*N* = 2) were treated at day 28.

### Immunogenicity.

#### Antibody responses.

##### Pf circumsporozoite protein (PfCSP) and PfSPZ.

**Figure 3.**
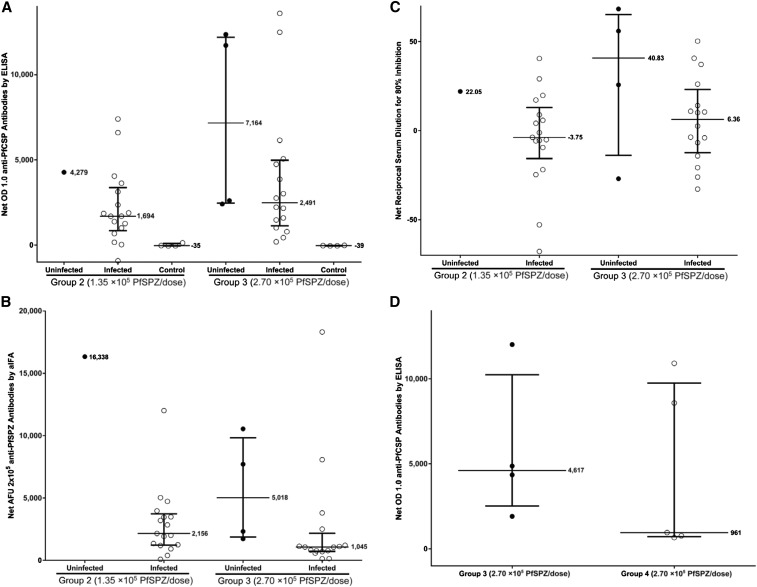

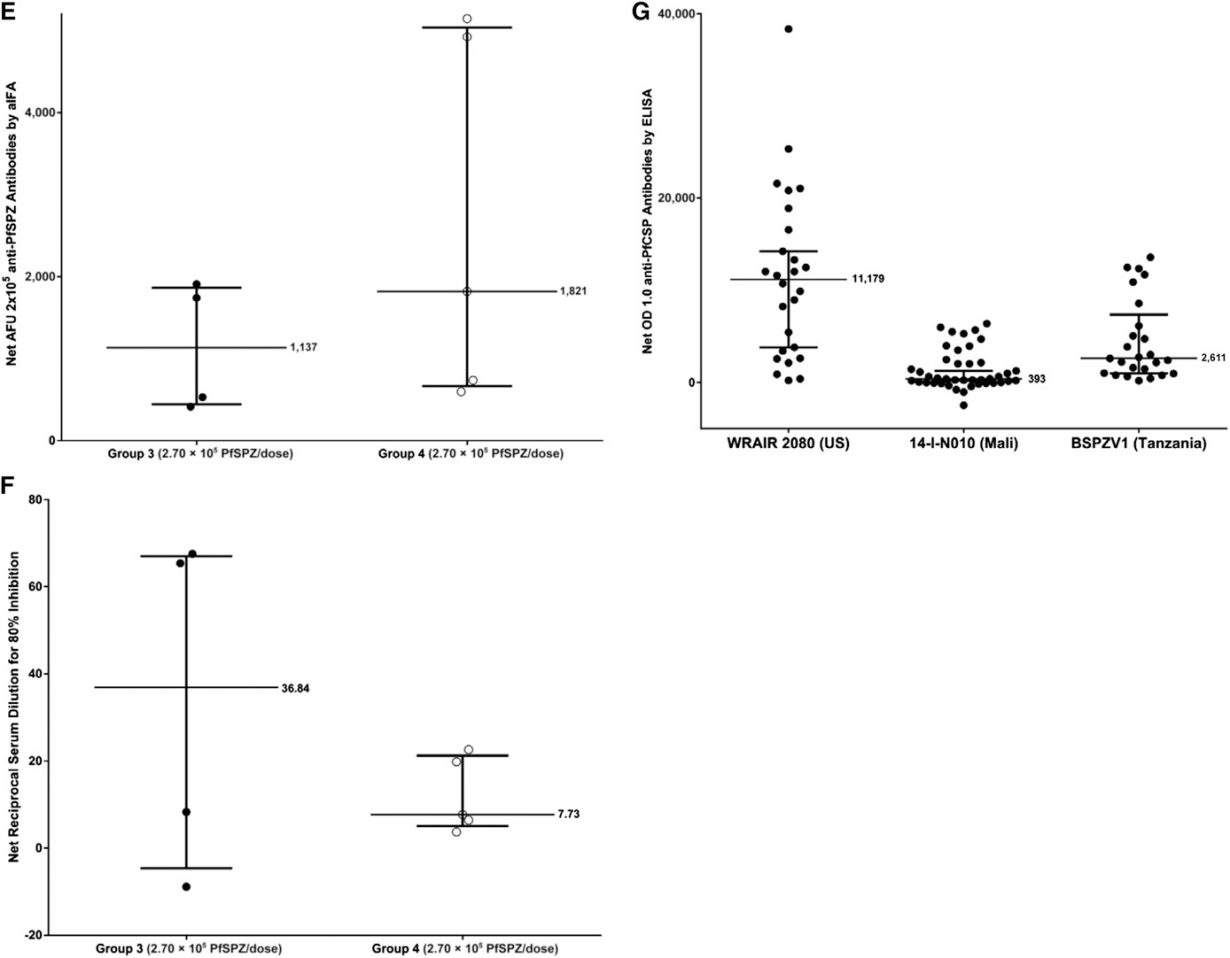
Antibody responses to *Plasmodium falciparum* Sporozoites (PfSPZ) and PfCSP before controlled human malaria infection (CHMI). For all assays, uninfected subjects are shown as filled (black) circles and infected subjects are open circles. For each of the defined subject groups, the interquartile ranges and the median values of response of subjects in each group are shown. Assessment of antibodies was performed in sera from subjects before immunization and before CHMI #1 (∼2 weeks after the last dose of PfSPZ Vaccine or normal saline [NS]) and/or CHMI #2 (∼24 weeks after last dose of PfSPZ or NS) (**A**, **D**). Antibodies to PfCSP by ELISA are reported as net optical density (OD) 1.0 (the difference in OD 1.0 between pre-CHMI and preimmunization sera). (**B**, **E**) Antibodies to PfSPZ by aIFA are reported as net AFU 2 × 10^5^, the reciprocal serum dilution at which the fluorescent units were 2 × 10^5^ (AFU 2 × 10^5^) in pre-CHMI minus preimmunization sera. (**C**, **F**) Results of inhibition of sporozoite invasion (ISI) assay are reported as serum dilution at which there was 80% reduction of the number of PfSPZ that invaded a human hepatocyte line (HC-04) in the presence of pre-CHMI as compared with preimmunization sera from the same subject. Panels **A–C** show groups 2 (five doses of 1.35 × 10^5^ PfSPZ) and 3 (five doses of 2.7 × 10^5^ PfSPZ) before short-term CHMI (2 weeks after the last dose of PfSPZ or NS) and panels **D–F** show those volunteers in Groups 3 (five doses of 2.7 × 10^5^ PfSPZ) and 4 (five doses of 2.7 × 10^5^ PfSPZ) who underwent long-term CHMI (24 weeks after the last dose of PfSPZ). Panel **G** shows net optical density (OD) 1.0 anti-PfCSP antibodies by an enzyme-linked immunosorbent assay (ELISA) comparing vaccinated Tanzanian volunteers to volunteers in other trials receiving the same regimen. After five doses of 2.70 × 10^5^ PfSPZ/dose, volunteers in bagamoyo sporozoite vaccine 1 (BSPZV1) (*N* = 25) had a 4.3-fold lower median net OD 1.0 than those in the U.S.-based clinical trial Walter Reed Army Institute of Research (WRAIR) 2080 (*N* = 26) but a 6.6-fold higher median OD 1.0 than volunteers in 14-I-N010 in Bamako, Mali (*N* = 42), where malaria transmission rates are higher. There was a significant difference between the results for WRAIR 2080 vs. BSPZV1 (*P* = 0.0012), WRAIR 2080 vs. 14-I-N010 (*P* < 0.0001), and even 14-I-N010 vs. BSPZV1 (*P* = 0.002) (two-tailed *t-*test). AFU = arbitrary fluorescence units; aIFA = antibodies by immunofluorescence assay.

Antibodies against PfCSP by ELISA 1), PfSPZ by aIFA 2), and PfSPZ by ISI 3) in sera taken 2 weeks after the last vaccine dose and just before CHMI (20–23 days after the last dose) for Groups 2 (CHMI #1) and 3 (CHMI #2) are in Figure 3A–C. The median responses and those uninfected and infected by qPCR are shown.

For all three assays, median antibody responses before first CHMI were higher in uninfected than in infected vaccinees. There was a significant difference in median net aIFA responses between infected and uninfected volunteers in Group 3 before CHMI #1 (*P* = 0.0499, Wilcoxon Rank-Sum Test), but not PfCSP (*P* = 0.290) or for ISI (*P* = 0.249).

In sera collected before CHMI #3 (170–171 days after the last vaccine dose), antibodies by the three assays for Group 4 and for the four volunteers in Group 3 uninfected in CHMI #1 who underwent CHMI #2 are in Figure 3D–F. All data appear in Supplemental Table 12.

After the fifth dose, in the PfCSP ELISA, volunteers were considered to have seroconverted if their net OD 1.0 and OD 1.0 ratio calculated, respectively, by subtracting or dividing by the prevaccination antibody OD 1.0, were ≥ 50 and ≥ 3.0. By these criteria, 15/18 volunteers (83%) in Group 2, 20/20 (100%) in Group 3, and 5/5 (100%) in Group 4 seroconverted, median net OD 1.0 of positives of 1,189, 2,685, and 961, and median OD 1.0 ratio of positives of 11.50, 21.15, and 37.83, respectively (Supplemental Table 13). In the aIFA, volunteers with a net arbitrary fluorescence unit (AFU) 2 × 10^5^ of ≥ 150 and a ratio of post- to pre-AFU 2 × 10^5^ of ≥ 3.0 were considered positive (Supplemental Table 13). By these criteria, 17/18 volunteers (94%) in Group 2, 18/20 (90%) in Group 3, and 5/5 (100%) in Group 4 seroconverted, median net OD 1.0 of positives of 2,844, 1,165, and 1,820, and median OD 1.0 ratio of positives of 1,193.00, 552.88, and 224.86, respectively (Supplemental Table 13). For the ISI, volunteers with a net ISI activity of ≥ 10% and a ratio of post- to pre-ISI activity of ≥ 3.0 were considered positive. By these criteria, 3/18 volunteers (17%) in Group 2, 8/20 (40%) in Group 3, and 3/5 (60%) in Group 4 were positive, median net OD 1.0 of positives of 22.05, 38.92, and 12.44, and median OD 1.0 ratio of positives of 19.79, 12.53, and 13.44, respectively (Supplemental Table 13).

##### Other antigens.

Two weeks after the fifth dose in Group 2 (1.35 × 10^5^ PfSPZ) and groups 3 and 4 (2.7 × 10^5^ PfSPZ), there were antibodies to PfCSP in 15/18 and 25/25 subjects, respectively. Ten of 25 volunteers immunized with 2.7 × 10^5^ PfSPZ made antibodies to Pf apical membrane antigen 1 and 4–16% responded to PfCelTOS, PfMSP5, PfMSP1, or Pf erythrocyte binding antigen 175 (PfEBA175) (Supplemental Table 14). The presence of antibodies, albeit at low incidence, against proteins first expressed in late liver stages (PfMSP1 and PfEBA175) was unexpected; results were confirmed by repeating the assays. No antibody responses were associated with protection.

##### T-cell responses.

T cells against liver-stage malaria parasites in mice and nonhuman primates immunized with radiation-attenuated SPZ mediate protection^9,29–31^ and it is likely this is the case in humans.^12^ CD8 and CD4 T-cell responses generally peak after the first vaccination with PfSPZ Vaccine.^13^ In this trial, T-cell responses were measured before immunization, 2 weeks after the first and 2 weeks after the final immunization in Group 2 (1.35 × 10^5^ PfSPZ). For technical reasons (loss of viability), the other groups could not be studied.

**Figure 4.**
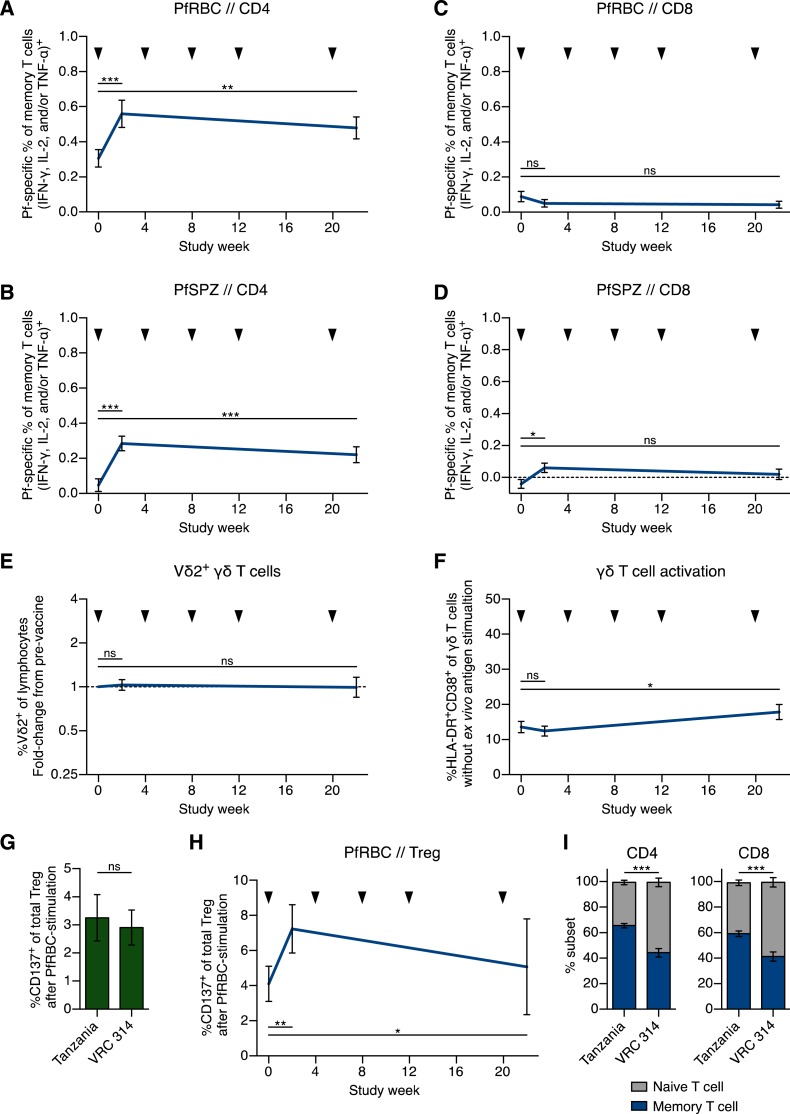
*Plasmodium falciparum* Sporozoites (PfSPZ)–specific T-cell responses in vaccine recipients receiving 1.35 × 10^5^ PfSPZ. (**A–D**) PfSPZ-specific T-cell responses. Frequency of cytokine-producing memory CD4 T cells responding to (**A**) PfRBC or (**B**) PfSPZ. Throughout, “naïve T cell” refers to cells that co-express CCR7 and CD45RA, and “memory T cell” refers to all other T cells. Frequency of cytokine-producing memory CD8 T cells responding to (**C**) PfRBC or (**D**) PfSPZ. Results are the percentage of memory T cells producing interferon gamma, interleukin 2, and/or tumor necrosis factor alpha following stimulation minus the percentage of cells following control stimulation. (**E**) Frequency of the Vδ2^+^ subfamily of γδ T cells of total lymphocytes. Results are expressed as fold-change from the prevaccine frequency. (**F**) γδ T-cell activation in vivo. Data are the percentage of memory γδ T cells expressing HLA-DR and CD38 as measured on PBMCs following incubation with control stimulation (vaccine diluent). (**G**) Prevaccine frequency of PfRBC-specific Tregs in Tanzania compared with malaria-naïve U.S. subjects from the Vaccine Research Center (VRC) 314 study. (**H**) Frequency of PfRBC-specific Treg. Results are the percentage of CD4^+^Foxp3^+^CD25^+^CD127^−^ T cells expressing CD137 (also known as 4-1BB) after stimulation with Pf red blood cell (PfRBC) minus the percentage of cells following stimulation with uninfected RBC. (**I**) Percentage of total CD4 (left) or CD8 (right) T cells that are naïve (gray bar; CCR7^+^CD45RA^+^) or memory (blue bar; not CCR7^+^CD45RA^+^) phenotype assessed prevaccination in all 48 subjects vaccinated in Tanzania or in 14 healthy U.S. subjects from the VRC 314 study.^13^ For **A**–**F** and **H**, *N* = 24, and statistical difference was measured by using the Wilcoxon matched-pairs signed rank test. For **G** and **I**, statistical difference was measured by using the Mann–Whitney *U* test. *P* values are reported as not significant (ns), < 0.05 (*), < 0.01 (**), or < 0.001 (***). Data are mean ± SEM. Time points are prevaccine, 2 weeks after the first vaccination, and 2 weeks after the final vaccination. Black arrowhead designates PfSPZ Vaccine administration. This figure appears in color at www.ajtmh.org.

After the first vaccination, the percent of Pf red blood cell (PfRBC)-specific and PfSPZ-specific cytokine-producing memory CD4 T-cell responses increased by 0.25 ± 0.06 (mean ± SEM) and 0.24 ± 0.04, respectively (Figure 4A, B). Throughout, “naïve T cell” refers to cells that co-express CCR7 and CD45RA, and “memory T cell” refers to all other T cells. After the final vaccination, at week 22, the CD4 T-cell responses were above prevaccine responses by 0.17 ± 0.05 and 0.18 ± 0.05% points, respectively. These responses were lower than after the same immunization regimen in malaria-naïve U.S. adults.^10^

PfRBC-specific CD8 T cells were not significantly above the prevaccine levels, and PfSPZ-specific CD8 T cells were slightly above background (Figure 4C, D); responses were lower than in U.S. adults.^10,12^

In contrast to other PfSPZ Vaccine trials,^10,12–14^ there was negligible change in the frequency of circulating γδ T cells (Figure 4E) or activation as measured by change in expression of the activation markers HLA-DR and CD38 following immunization (Figure 4F). To identify potential explanations for lower cellular immune responses in Tanzanians, we examined frequency of T regulatory (Treg) cells (CD4^+^Foxp3^+^CD25^+^CD127^−^) expressing the activation marker CD137 (also known as 4-1BB)^32^ after stimulation with PfRBC. There was no difference in prevaccine frequency of PfRBC-specific Tregs in the Tanzanians as compared with Americans^10^ (Figure 4G). Consistent with CD4 and CD8 T-cell responses, PfRBC-specific Tregs were highest after first immunization (Figure 4H). Last, the prevaccine frequency of total memory T cells relative to total naïve T cells was significantly higher than in Americans (Figure 4I).

## DISCUSSION

To our knowledge, this was the first assessment of the VE of a malaria vaccine in Africa against CHMI. *Plasmodium falciparum* SPZ Vaccine was well tolerated and safe but less immunogenic and protective in Tanzanian men than in U.S. volunteers.

In our studies, all 18 controls became infected. Four of 20 (20%) recipients of five doses of 2.7 × 10^5^ PfSPZ did not become infected after homologous CHMI by DVI 3 weeks after the last immunization. By contrast, 12/13 (92.3%) volunteers in the United States who received five doses of 2.7 × 10^5^ PfSPZ were protected after homologous CHMI by mosquito bite 3 weeks after the last vaccine dose.^11^ When the four uninfected Tanzanian volunteers underwent repeat homologous CHMI at 24 weeks after the last dose, all four (100%) were protected. In the United States, Seven of 10 previously protected volunteers were protected when they underwent homologous CHMI at 24 weeks^11^ and all five volunteers in the United States who were protected at 21 weeks after the last immunization (four doses of 2.7 × 10^5^ PfSPZ) were protected against repeat mosquito-administered CHMI at 59 weeks.^12^ This could be due to boosting by the small numbers of PfSPZ administered during the CHMI, or is more likely due to the fact that in these protected individuals, the protective immune responses induced by immunization were sustained.

The same exact immunization regimen was assessed for VE against intense field transmission of heterogeneous Pf in Mali. Vaccine efficacy against infection with Pf on TBS was 52% by time to event and 29% by proportional analysis during 24 weeks after the last vaccine dose.^14^ This was higher than the VE by proportional analysis against homologous CHMI in Tanzania. In Tanzania, there was a significant delay in the onset of parasitemia after CHMI at 3 and 24 weeks in subjects who received five doses of 2.7 × 10^5^ PfSPZ and were not fully protected (Figure 2B–D). Nonetheless, the proportional analysis suggests that homologous CHMI by DVI of a 100% infectious dose of homologous PfSPZ Challenge is at least as rigorous as a test of VE and potentially more rigorous than intense field transmission of heterogeneous Pf.

Vaccine-induced antibody and T-cell responses in the Tanzanians were lower than in malaria-naïve Americans who received the exact same dosage regimen. Two weeks after the last dose, the median antibody responses to PfCSP, the major protein on the surface of PfSPZ, were 4.3 times lower in the Tanzanians than those in Americans (*P* = 0.0012, Student’s *t*-test, 2-tailed),^11^ but significantly higher than in Malians who received the same immunization regimen (*P* = 0.002)^14^ (Figure 3G).

The T-cell responses were also lower than in Americans^10,12^ (Figure 4), but this could only be assessed in PBMCs from individuals who received the lower dose (five doses of 1.35 × 10^5^ PfSPZ), not in the individuals who received the higher dose (five doses of 2.7 × 10^5^ PfSPZ), the group that had sustained protection for 24 weeks. Thus, it is possible that had PBMCs from the higher dose group been assessed, responses would have been comparable to the responses in nonimmune Americans. The Tanzanians who were assessed had a significantly higher proportion of total memory T cells compared with total naïve T cells at the baseline than did the Americans. This higher frequency of memory cells compared with naïve cells may explain the lower immunogenicity due to less available naïve cells for expansion during the vaccinations. Moreover, the greater frequency of non-Pf–specific memory T cells may compete for infected cell contacts during pathogen surveillance.^33^ These data suggest that PfSPZ Vaccine immunogenicity may be dependent on cumulative history of Pf exposure. Another explanation is that an activated immune microenvironment in the Tanzanians as compared with the Americans reduced immune responses.^34^ Helminth infections have been associated with reduced immune responses to malaria^35^; however, the paucity of helminth infections in this population does not support helminth infection as a cause of the reduced immune responses.

There were no differences between vaccine and NS placebo recipients in regard to vaccine tolerability or AEs; 97.1% of the DVI administrations were rated painless and no volunteer experienced any local AE. Systemic AEs, most commonly headache, were mild, infrequent, and of short duration, with a similar frequency in NS controls as in vaccinees (no statistically significant differences in rates).

Among the controls, 16 of 18 were positive for Pf by TBS after CHMI. However, all 18 were positive by qPCR. This is consistent with findings in Gabon after CHMI.^21^ It is likely that preexisting asexual blood stage immunity limits Pf replication in some individuals. Thus, they never reach the threshold for detection by TBS. In our CHMI studies in Bagamoyo, we now use qPCR to confirm positive TBS, and retrospectively or in real time, assess parasitemia in all volunteers by qPCR.

We propose that increasing the numbers of PfSPZ per dose and altering intervals between doses will lead to overcoming the downregulation of humoral and cell-mediated immunity most likely because of previous exposure to Pf and thereby increase immune responses to PfSPZ Vaccine and VE. We also hypothesize that immune responses in younger, less malaria-exposed individuals will be of greater magnitude than those in adults.

## Supplementary Material

Supplemental tables and figure
