# Supplementary material for: Safety, Immunogenicity, and Protective Efficacy against Controlled Human Malaria Infection of Plasmodium falciparum Sporozoite Vaccine in Tanzanian Adults
Source: Am J Trop Med Hyg. 2018 Jun 25;99(2):338–49. doi: 10.4269/ajtmh.17-1014 (PMC6090339; doi:10.4269/ajtmh.17-1014)
Supplement: Supplementary file 1 [file tpmd171014.SD1.pdf]

Supplemental Data

Table S1: Assessment of volunteer understanding
All questions are answered true or false:

1. The main purpose of the study is to see if the experimental vaccine is safe.
2. I have the right to discontinue my participation at any time.
3. Everyone in each group will receive the same dosage of the malaria vaccine
4. Study vaccine will be given intravenously.
5. The duration or participation in the study is different for each volunteer group.
6. There may be side effects from the experimental vaccine products that are unknown.
7. I may be asked to return for additional clinic visits if any test results need to be clarified with repeat testing.
8. If I have any questions/concerns after normal clinic business hours, I should not "bother" the staff, and should call the next day.
9. All volunteers must not donate blood or participate in any other research during the study period.
10. All groups will participate in the malaria challenge two times.
11. Volunteers will be asked to keep a diary cards to record side effects after each vaccination.
12. If I develop any malaria symptoms I should wait until my next visit to tell the nurse.
13. lf I miss a scheduled visit, I can make it up at any time.
14. After the study concludes, if some of my remaining collected blood samples are required to be used in the future for other analysis, investigators will need approval from Ethical Committee.
15. After each vaccination I will be asked to stay for 48 hours in the clinical trial unit for observation.
16. Strict adherence to the protocol schedule is not that important.
17. The study vaccine will definitely protect those who receive it from getting malaria.
18. Volunteers who participate in the malaria challenge will stay at the clinical trial unit for observation for 48 hours and later on day 9 they will be asked to stay again at the unit for a maximum period of 13 days.
19. Volunteers who develop serious side effects must inform a study doctor promptly.
20. This is the first study conducted in a malaria endemic area in Africa in which PfSPZ (strain NF54) will be administered via the IV route for immunization and CHMI.

Any volunteer unable to answer 100% of these questions correctly on the first or second attempt will be excluded from the study.

Table S2: Inclusion Criteria

Healthy male aged between 18 – 35 years.

Good health status based on history and clinical examination.

Long term or permanent resident in or near Dar-es-Salaam.

Able and willing to complete the study visit schedule over the one year follow up period, including the hospitalizations required for protocol compliance.

Able and willing to complete the informed consent process conducted in English.

Demonstrate understanding of the study and procedures by answering 20 questions from the Protocol & Study Procedures Understanding Checklist correctly with a maximum of two attempts.

Agrees to inform study doctor of medical conditions and contra-indications for participation in the study.

Agrees to provide contact information to the study team for a household member who will serve as an emergency contact during trial participation.

Willing to be attended by a study doctor and take medications, which may be prescribed by a study doctor, during study participation.

Reachable (24/7) by mobile phone during the whole study period.

Agrees not to participate in another study during the study period.

Agrees not to donate blood during the study period.

Willing to undergo HIV, hepatitis B and hepatitis C testing.

Willing to undergo controlled human malaria infection (CHMI).

Supplemental Table S3: Exclusion Criteria

History of malaria in the past 5 years.

Positive for malaria by thick blood smear at screening.

Plans to travel outside the Dar-es-Salaam or Coast Region in first 12 months of the study.

Previous receipt of an investigational malaria vaccine.

Antibodies to parasites or selected parasite protein(s) above acceptable cut off established for the site.

History of arrhythmias or prolonged QT-interval or other cardiac disease or clinically significant abnormalities in electrocardiogram (ECG) at screening.

History or indication of a history of drug or alcohol abuse interfering with normal social function.

Use of chronic immunosuppressive drugs, antibiotics, or other immune modifying drugs within three months of study enrollment (inhaled and topical corticosteroids are allowed).

Ongoing condition that could interfere with the interpretation of the study results or compromise the health of the volunteer.

History of diabetes mellitus or cancer.

Body Mass Index (BMI) below 18 or above 30 kg/m^2.

Any clinically significant deviation from the normal range in biochemistry or hematology blood tests or in urine analysis or electrolytes.

Positive HIV, Hepatitis B virus or Hepatitis C virus tests.

Participation in any other clinical study within 30 days prior to study enrollment.

Known hypersensitivity, allergy, or other contra-indications to Coartem® or Malarone® including treatment taken by the volunteer that interferes with Coartem® or Malarone®.

Any confirmed or suspected immunosuppressive or immunodeficient condition, including asplenia.

Heterozygous or homozygous for sickle cell or homozygous for alpha thalassemia.

Glucose-6-phosphate dehydrogenase deficiency

Psychiatric condition that precludes compliance with the protocol; past or present psychoses; disorder requiring lithium; or within five years prior to enrollment, history of suicide plan or attempt.

Any medical, psychiatric, social condition, or occupational reason that, in the judgment of the investigator, is a contraindication to protocol participation or impairs the volunteer's ability to give

informed consent, increases the risk to the volunteer because of participation in the study, affect the ability of the volunteer to participate in the study or impair interpretation of the study data.

History of 3 or more other immunizations within the six months before administration of the first dose of vaccine.

Clinically active tuberculosis

Table S4: Solicited Local and Systemic AEs

Local	Systemic
Bruising	Fever
Redness	Headache
Induration (hardness)	Malaise
Swelling	Fatigue
Pain	Myalgia
Tenderness	Arthralgia
	Nausea
	Vomiting
	Chills
	Diarrhea
	Abdominal pain
	Chest pain
	Palpitations
	Shortness of breath

Table S5: ELISA measuring antibodies against proteins expressed in sporozoites (PfCSP, PfSSP2/TRAP, PfCelTOS, PfMSP5, PfAMA1), early liver stages (PfEXP1 and PfSLSA1) and late liver/blood stages (PfMSP1and PfEBA175)

	**Recombinant Protein Details**
PfCSP	The recombinant *P. falciparum* (Pf) circumsporozoite protein (rPfCSP, 3D7 strain) is a 48 kDa protein with an N-terminal sequence SLGEND, approximately 299 amino acids in length, without the signal sequence. The 1st amino acid is residue 50 at the N-terminus and comprising both the the N- and C-portions with 22 NANP and 4 NVDP repeats, without the last 13 amino acids of the C-terminal portion. GenBank Accession No.: ADF48458.1 (Protein Potential).
PfSSP2/ TRAP	The recombinant Pf sporozoite surface protein 2 (recombinant fragment of PfSSP2, 3D7) is a 27 kDa protein. The rPfSSP2 lacks the hydrophobic regions at the amino terminus (22 aa) and the C-terminus (63 aa). The sequence selected for expression is the entire extracellular domain of PfSSP2, which includes the A-type domain (of von Willebrand factor) and the type 1 repeat of thrombospondin (TSR). GenBank Accession No.: AAC18657.1 (Protein Potential).
PfMSP5	The recombinant Pf merozoite surface protein-5 is a 40-kDa protein that is located on the merozoite surface and is non-covalently associated with merozoite surface protein 1 (MSP1) complex shed from the surface at erythrocyte invasion. GenBank Accession No: AAF12722.1 (Ross Coppel, Monash University, Australia) (11, 12).
PfCelTOS	The recombinant Pf cell-traversal protein for ookinetes and sporozoites (rPfCelTOS) is an 18 kDa protein with an N-terminal sequence FRGNNG. It is approximately 151 amino acids in length, with the first amino acid being residue 25 at the N-terminus without the hydrophobic signal sequence. GenBank Accession No.: BAD97684.1 (Protein Potential).
PfMSP1	PfMSP142 (EcMSP142-3D7, Lot# WRAIR11150, 0.99 mg/mL) is a recombinant of the C-terminal 42-kDa portion of the merozoite surface protein-1 (PfMSP1) from the 3D7 strain of Pf expressed, refolded, and purified at the Walter Reed Army Institute of Research (WRAIR) Pilot Bioproduction Facility. GenBank Accession Number: ABS84655.1 (WRAIR).
PfAMA1	The recombinant Pf apical membrane antigen-1 PfAMA1 (rPfAMA1-3D7, Lot# MV-1187 Final Bulk Protein, 1.00 mg/mL). The precursor of AMA1 is processed proteolytically, to cleave away the pro-sequence, converting the protein into a 66 kDa form, which allows the merozoite relocalization. GenBank Accession No: AAN35928.1 (Carole Long, NIAID, NIH).
PfEBA175	The recombinant Pf erythrocyte binding antigen 175 (rPfEBA175 RII, Lot# 321-0904-007, 2.38 mg/mL) region II with an N-terminal sequence GRQTSS, approximately 616 amino acids in length. GenBank Accession No.: AAF72186.1 (Protein Potential).
PfLSA1	PfLSA1 (recombinant 52-kDa Lot# 061907, 0.50 mg/mL). The recombinant Pf liver stage antigen-1, PfLSA1 is a 20-kDa protein with an N-terminal sequence KENKLN, approximately 259 amino acids in length, the first amino acid being residue 28 at the N-terminus and truncated at residue 286 at the C-terminus. GenBank Accession No.: AAW78332.1 (Protein Potential).
PfEXP1	Pf exported protein-1 (rPfEXP1, 23-kDaLot# 010507, 1.00 mg/mL).<br>The recombinant Pf exported protein-1 is a 23 kDa protein with an N-terminal sequence SLAEKT and is approximately 143 amino acids in length, without the hydrophobic signal sequence removed. GenBank Accession No.: CAA28735.1 (Protein Potential).

**Assay Details**									
Pf Proteins	**CSP**	**SSP2**	**MSP5**	**CelTOS**	**MSP1**	**AMA1**	**EBA175**	**LSA1**	**EXP1**
Coating antigen concentration in 50 µL per well	2.0 µg /mL	1.5 µg /mL	2.0 µg /mL	1.5 µg /mL	1.5 µg /mL	2.0 µg /mL	1.0 µg /mL	1.0 µg /mL	0.5 µg /mL
Washing Buffer	1X Imidizole-based wash solution containing 2 mM imidazole, 160 mM NaCl, 0.02% Tween 20, 0.5 mM EDTA (3 times after each incubations)								
Blocking buffer composition (diluent)	1% Bovine Serum Albumin (BSA) blocking buffer (KPL)								
	1% milk	5% milk	1% milk	5% milk	5% milk	1% milk	5% milk	1% milk	1% milk
Serum	1:100 starting dilution and three fold serial dilution in triplicate								
Secondary ab	Peroxidase labeled goat anti-human IgG (KPL)								
Secondary ab µg/mL	0.1	0.1	0.1	0.1	0.05	0.1	0.05	0.2	0.1
Substrate	ABTS Peroxidase substrate								
Substrate incubation period mins)	75	60	75	60	60	75	60	60	50

Table S6. ELISA to measure human antibodies against PfEXP1 to detect recent malaria exposure in sera for volunteer screening. Volunteers whose serum showed an OD > 1 at a 1:600 dilution were considered malaria-exposed. 31/137 (29%) of subjects were excluded, because of a positive result. Serum acquired from a blood bank in Kenya was used as a positive control and the results expressed as the reciprocal serum dilution at which OD = 1 (OD 1.0).

**OD at Dilution 1:300X**	**OD at Dilution 1:600X**	**Reactivity with OD > 1 at 1:600X**	**Positive Control (OD = 1)**
**2.566**	**1.815**	**Positive**	
**3.185**	**2.812**	**Positive**	
0.255	0.167	Negative	
1.217	0.783	Negative	
0.229	0.36	Negative	
0.305	0.188	Negative	
0.462	0.289	Negative	
0.412	0.266	Negative	**15,704**
1.205	0.705	Negative	
1.069	0.586	Negative	
0.213	0.148	Negative	
0.821	0.493	Negative	
0.22	0.15	Negative	
0.186	0.134	Negative	
0.66	0.385	Negative	
1.189	0.723	Negative	
**3.079**	**2.788**	**Positive**	
**3.357**	**3.289**	**Positive**	
**1.872**	**1.179**	**Positive**	
**3.382**	**3.245**	**Positive**	
**3.167**	**2.796**	**Positive**	
**2.481**	**1.711**	**Positive**	
**2.778**	**2.166**	**Positive**	
**3.248**	**3.012**	**Positive**	
**2.778**	**1.99**	**Positive**	
**3.168**	**2.929**	**Positive**	
**1.733**	**1.066**	**Positive**	
**3.388**	**3.279**	**Positive**	
**3.7**	**3.662**	**Positive**	
**3.157**	**2.824**	**Positive**	
**3.317**	**3.061**	**Positive**	
0.329	0.209	Negative	
0.196	0.149	Negative	
0.336	0.211	Negative	
0.243	0.157	Negative	
0.37	0.217	Negative	**10,760**
0.239	0.161	Negative	
0.476	0.284	Negative	
0.791	0.406	Negative	
0.13	0.108	Negative	
0.828	0.469	Negative	
1.072	0.572	Negative	
0.139	0.133	Negative	
0.475	0.281	Negative	
0.106	0.096	Negative	
1.462	0.884	Negative	
1.571	0.911	Negative	
0.16	0.142	Negative	
0.404	0.235	Negative	
1.394	0.871	Negative	
0.144	0.119	Negative	
0.423	0.274	Negative	
0.54	0.32	Negative	
0.935	0.564	Negative	
0.8	0.428	Negative	

1.289	0.754	Negative	
0.416	0.212	Negative	
1.126	0.575	Negative	
0.121	0.11	Negative	
0.448	0.255	Negative	
0.622	0.379	Negative	
0.661	0.401	Negative	
0.682	0.393	Negative	
0.471	0.283	Negative	
0.497	0.348	Negative	
0.155	0.123	Negative	
0.257	0.159	Negative	
1.605	0.852	Negative	
0.413	0.263	Negative	
1.226	0.785	Negative	
0.413	0.242	Negative	
0.362	0.229	Negative	
0.539	0.32	Negative	
0.462	0.273	Negative	
0.163	0.125	Negative	
0.161	0.123	Negative	
1.007	0.562	Negative	
0.251	0.162	Negative	
1.404	0.801	Negative	
0.241	0.165	Negative	
0.161	0.123	Negative	
0.278	0.164	Negative	
0.208	0.147	Negative	
1.095	0.595	Negative	
0.211	0.151	Negative	
0.381	0.211	Negative	
0.523	0.308	Negative	
0.805	0.500	Negative	
0.292	0.209	Negative	
0.123	0.107	Negative	
0.251	0.189	Negative	
0.269	0.182	Negative	
1.284	0.727	Negative	
0.598	0.381	Negative	
0.195	0.149	Negative	
0.369	0.215	Negative	
1.133	0.587	Negative	
0.436	0.227	Negative	
1.781	0.991	Negative	
**3.257**	**2.84**	**Positive**	**13,395**
**3.265**	**2.938**	**Positive**	
**3.587**	**3.588**	**Positive**	
0.182	0.143	Negative	
0.143	0.122	Negative	
0.406	0.301	Negative	
1.102	0.618	Negative	
0.701	0.42	Negative	
1.739	0.942	Negative	
0.705	0.435	Negative	
0.6	0.362	Negative	
1.085	0.677	Negative	
**3.123**	**2.682**	**Positive**	**12,981**
1.045	0.688	Negative	
1.025	0.601	Negative	
0.592	0.392	Negative	
0.816	0.46	Negative	
1.202	0.709	Negative	
1.359	0.807	Negative	
**3.495**	**3.430**	**Positive**	**20,284**
**2.171**	**1.414**	**Positive**	
**2.992**	**2.232**	**Positive**	

**1.839**	**1.225**	**Positive**	
**3.194**	**2.778**	**Positive**	
**3.410**	**3.247**	**Positive**	
**2.785**	**1.992**	**Positive**	
**3.306**	**2.964**	**Positive**	
**1.536**	**1.007**	**Positive**	
**1.953**	**1.353**	**Positive**	
0.484	0.401	Negative	
0.517	0.506	Negative	
1.369	0.858	Negative	
1.220	0.678	Negative	
1.136	0.738	Negative	
1.076	0.733	Negative	
0.595	0.439	Negative	
1.198	0.729	Negative	
1.195	0.766	Negative	

Table S7. Flow cytometry panels for T cell studies.

#	Detector	Fluorophore	Panel 1 Stim: PfRBC/uRBC	Panel 2 Stim: PfSPZ/HSA	Panel 3 Stim: PfRBC/uRBC
1	B515	FITC	Ki-67		TCR-Vδ1
2	B710	Cy5.5PerCP	TCR Vα7.2		
3	V440	BV421			CD127
4	V510	Aqua/BV510	Viability/CD14	Viability/CD14	Viability/CD14
5	V570	BV570			
6	V605	BV605	IL-2	IL-2	CD25
7	V650	BV650	TNF-α	TNF-α	
8	V710	BV711	CD56	HLA-DR	
9	V750	BV750			
10	V800	BV785	CD4	CD4	CD4
11	R660	APC/Ax647	IFN-γ	IFN-γ	Foxp3
12	R710	Ax680	CCR7	CCR7	CCR7
13	R780	Cy7APC	CD3	CD3	CD3
14	G560	PE	CXCR6		CD137
15	G610	CF594PE	TCR-γδ	TCR-γδ	TCR-γδ
16	G660	Cy5PE	CD161	CD38	TCR-Vδ2
17	G710	Cy5.5PE	CD45RA	CD45RA	CD45RA
18	G780	Cy7PE	CD8	CD8	CD8

Table S8 Antibody clones and manufacturers.

Specificity	Fluorophore	Clone	Manufacturer	Catalog
CD3	Cy7APC	SP34.2	BD	557757
CD4	BV785	OKT4	BioLegend	317441
CD8	Cy7PE	RPA-T8	BioLegend	301012
CD14	BV510	M5E2	BioLegend	301842
CD25	BV605	BC96	BioLegend	302632
CD38	Cy5PE	HIT2	BD	555461
CD45RA	Cy5.5PE	MEM-56	Invitrogen	MHCD45RA18
CD127	BV421	A019D5	BioLegend	351310
CD137	PE	4B4-1	BD	555956
CD161	Cy5PE	DX12	BD	551138
CCR7	Ax680	150503	VRC	--
CXCR6	PE	K041E5	BioLegend	356004
HLA-DR	BV711	L243	BioLegend	307644
TCR Vα7.2	PerCPCy5.5	3C10	BioLegend	351710
TCR-γδ	CF594PE	B1	BD	562511
TCR-Vδ1	FITC	TS8.2	ThermoScientific	TCR2730
TCR-Vδ2	Cy5PE	B6	VRC	--
IFN-γ	APC	4S.B3	BioLegend	502512
IL-2	BV605	MQ1-17H12	BioLegend	500331
TNF-α	BV650	MAb11	BioLegend	502937
Ki-67	FITC	B56	BD	556026
Foxp3	Ax647	259D	BioLegend	320214

Table S9. Prepatent period and parasite density for volunteers by group by TBS and qPCR.

	Group 2 (1.35x10^5 PfSPZ)	Group 2 Controls	Group 3 (2.7x10^5 PfSPZ)	Group 3 Controls	Group 4 (2.7x10^5 PfSPZ)	Group 4 Controls
Underwent and completed CHMI	18	6	20	6	5	6
Number thick blood smear (TBS) positive	15	6	16	6	4	4
**Number of days to first positive TBS = pre-patent period by TBS**						
Mean ± SD (days)	14.2±1.5	13.7±3.2	14.2±1.5	13.4±1.4	15.1±1.3	11.7±0.7
Median (days)	14.0	12.5	14.0	13.0	15.3	12.0
Range (days)	12.2-17.7	10.1-18.7	12.3-18.6	12.0-15.6	13.6-16.3	10.7-12.2
**Number positive by qPCR (VE by proportional analysis)**						
Total	17 (5.6%)	6 (0%)	16 (20%)	6 (0%)	5 (0%)	6 (0%)
**Number of days to first positive by qPCR=pre-patent period by qPCR***						
Mean ± SD (days)	9.4±1.2	9.3±1.0	9.3±0.7	9.0±0.6	9.0±1.0	7.5±0.2
Median (days)	9.2	8.9	9.0	8.7	9.4	7.6
Range (days)	8.6-13.6	8.7-11.4	8.7-10.6	8.7-10.2	7.9-10.0	7.2-7.9
**Difference in days between pre-patent period by TBS and pre-patent period by qPCR**						
Mean ± SD (days)	5.2±1.4	4.5±3.0	4.9±1.2	4.4±1.0	5.8±0.6	4.2±0.8
Median (days)	4.9	3.7	4.8	4.3	6.0	4.6
Range (days)	2.9-8.4	1.4-9.3	3.3-8.3	3.3-5.6	5.0-6.4	3.0-4.7

*For CHMIs #1 and #2 sampling for qPCR began on day 8 after injecton of PfSPZ Challenge. For CHMI #3 sampling for qPCR began on day 7 after injection of PfSPZ Challenge.

Table S10. Prepatent period and parasite density for individuals by TBS and qPCR.

Volunteer #	Vaccine	Time to first qPCR sample (days)	Time to qPCR+ (days)	Time to TBS+ (days)	parasite density qPCR+ [Pf/µL]	parasite density TS+ [Pf/µL]	parasite density qPCR on day of TS+ [Pf/µL]	CHMI Group
067G2	1.35×10^5 PfSPZ	8.80	9.35	17.75	0.4	17.6	48.1	1
104G2	1.35×10^5 PfSPZ	8.80	9.23	14.18	0.5	6.5	21.0	1
117G2	1.35×10^5 PfSPZ	8.78	8.78	15.17	0.6	4.6	156.8	1
269G2	1.35×10^5 PfSPZ	8.87	13.75	negative	7.8			1
087G2	1.35×10^5 PfSPZ	9.31	9.31	14.23	0.1	72.0	49.3	1
078G2	1.35×10^5 PfSPZ	Nd	negative	negative				1
093G2	1.35×10^5 PfSPZ	8.80	10.71	negative	0.1			1
048G2	1.35×10^5 PfSPZ	8.82	9.26	12.21	0.2	24.0	22.5	1
139G2	1.35×10^5 PfSPZ	8.79	8.79	13.20	0.7	19.5	20.9	1
126G2	1.35×10^5 PfSPZ	8.81	8.81	13.69	1.4	68.6	88.4	1
198G2	1.35×10^5 PfSPZ	8.79	8.79	13.68	0.8	45.4	131.7	1
144G2	1.35×10^5 PfSPZ	8.60	8.60	13.46	0.6	46.3	125.9	1
179G2	1.35×10^5 PfSPZ	8.74	13.62	negative	0.4			1
156G2	1.35×10^5 PfSPZ	8.79	8.79	12.19	1.5	15.0	58.7	1
205G2	1.35×10^5 PfSPZ	8.73	9.16	16.58	0.2	10.2	16.5	1
274G2	1.35×10^5 PfSPZ	8.73	8.73	14.24	1.0	169.5	60.5	1
218G2	1.35×10^5 PfSPZ	8.73	9.16	15.09	0.4	1.2	24.3	1
228G2	1.35×10^5 PfSPZ	8.72	10.10	13.96	0.2	12.0	11.3	1
239G2	1.35×10^5 PfSPZ	8.86	8.86	13.75	0.6	19.5	73.8	1
281G3	2.7×10^5 PfSPZ	8.72	8.72	14.38	0.2	6.5	28.9	2
306G3	2.7×10^5 PfSPZ	8.72	8.72	12.31	0.2	134.3	110.6	2
310G3	2.7×10^5 PfSPZ	8.73	9.14	13.58	0.4	10.3	45.1	2
325G3	2.7×10^5 PfSPZ	8.70	10.27	15.18	1.4	310.1	159.5	2
335G3	2.7×10^5 PfSPZ	8.71	8.71	12.51	0.5	12.1	66.5	2
345G3	2.7×10^5 PfSPZ	8.70	10.11	15.15	0.1	36.1	203.3	2
351G3	2.7×10^5 PfSPZ	Nd	negative	negative				2
420G3	2.7×10^5 PfSPZ	8.75	8.75	14.02	0.1	14.7	31.8	2

495G3	$2.7x10^5$ PfSPZ	8.73	10.21	18.56	0.3	10.2	25.1	2
377G3	$2.7x10^5$ PfSPZ	8.70	9.13	13.25	0.4	35.2	76.1	2
445G3	$2.7x10^5$ PfSPZ	Nd	negative	negative				2
453G3	$2.7x10^5$ PfSPZ	Nd	negative	negative				2
383G3	$2.7x10^5$ PfSPZ	8.75	10.64	13.95	2.9	13.0	39.0	2
397G3	$2.7x10^5$ PfSPZ	8.74	9.15	15.66	0.3	13.9	29.2	2
401G3	$2.7x10^5$ PfSPZ	8.40	8.80	14.00	0.1	433.3	221.3	2
469G3	$2.7x10^5$ PfSPZ	8.52	10.04	14.37	0.7	38.9	30.8	2
473G3	$2.7x10^5$ PfSPZ	8.78	8.78	12.54	0.4	13.9	67.8	2
489G3	$2.7x10^5$ PfSPZ	8.72	8.72	13.61	0.2	10.9	28.9	2
512G3	$2.7x10^5$ PfSPZ	8.73	8.73	13.47	0.2	6.5	27.5	2
411G3	$2.7x10^5$ PfSPZ	Nd	negative	negative				2
563G4	$2.7x10^5$ PfSPZ	7.89	7.89	negative	0.5			3
526G4	$2.7x10^5$ PfSPZ	7.87	7.87	13.60	1.0	24.6	ND	3
538G4	$2.7x10^5$ PfSPZ	7.85	9.75	16.14	0.1	27.1	ND	3
551G4	$2.7x10^5$ PfSPZ	7.48	10.05	16.28	0.1	10.2	25.9	3
570G4	$2.7x10^5$ PfSPZ	7.66	9.40	14.40	0.3	5.6	19.3	3
054G2	normal saline	8.82	8.82	12.21	0.1	23.0	54.7	1
240G2	normal saline	8.73	8.73	10.11	2.5	3.7	6.2	1
160G2	normal saline	8.41	8.93	12.61	0.3	63.0	30.1	1
253G2	normal saline	8.48	11.39	16.22	0.1	4.7	0.7	1
290G3	normal saline	8.73	8.73	14.37	1.0	5.6	8.8	2
360G3	normal saline	8.70	8.70	12.52	0.4	4.7	106.8	2
430G3	normal saline	8.71	8.71	12.20	0.4	45.4	42.0	2
500G3	normal saline	8.72	10.23	15.61	0.1	27.8	73.8	2
590G5	control	8.83	8.84	12.28	1.1	28.0	46.0	1
581G5	control	8.83	9.37	18.70	0.6	46.0	46.0	1
603G5	control	8.71	8.71	12.04	0.1	10.2	4.5	2
616G5	control	8.72	8.72	13.45	0.1	13.0	20.2	2
655G5	control	7.62	7.62	13.17	0.1	12.1	27.8	3
624G5	control	7.85	7.85	negative	1.1			3
661G5	control	7.23	7.23	12.97	0.8	8.4	29.8	3
649G5	control	7.65	7.65	11.65	2.3	4.7	22.6	3
631G5	control	7.50	7.50	13.07	0.8	9.3	30.9	3

676G5	control	7.22	7.22	negative	4.6			3

ND=not determined

Table S11: Clinical characteristics of CHMI in thick smear positive volunteers.

	Total PfSPZ Vaccine	Normal Saline and Infectivity Controls	P values – vaccine vs. controls
No. of volunteers thick blood smear positive	35	16	
Asymptomatic	20 (57%)	9 (56%)	>.05
Headache	11 (31%)	7 (44%)	>.05
Fever	9 (26%)	0	0.043
Abdominal pain	1 (2.9%)	0	>.05
Arthralgia	0	1 (6.3%)	>.05
Chills	0	0	>.05
Diarrhea	0	1 (6.3%)	>.05
Fatigue	0	1 (6.3%)	>.05
Malaise	3 (8.6%)	0	>.05
Myalgias	1 (2.9%)	1 (6.3%)	>.05
Nausea	1 (2.9%)	0	>.05
Vomiting	1 (2.9%)	0	>.05
Other	0	1 (6.3%)	>.05

Table S12: Raw immunological data from sera obtained from volunteers in BSPZV1. Volunteers 269 and 548 only received four doses; volunteers 188 and 548 underwent CHMI but dropped out of the study before protection could be determined. For this reason, their results were omitted when calculating medians.

Group	ID	PfCSP OD 1.0				ISI (80% Inhibition)				aIFA (PfSPZ)			
		Pre-Immune	2 weeks post-5th Dose	Pre-CHMI #1	Pre-CHMI #2	Pre-Immune	2 weeks post-5th Dose	Pre-CHMI #1	Pre-CHMI #2	Pre-Immune	2 weeks post-5th Dose	Pre-CHMI #1	Pre-CHMI #2
Group 2: 5 doses of 1.35x10^5 PfSPZ<br>CHMI at 19-21 Days	048	11	3141	.	.	7.33	47.85	.	.	0	5031	.	.
	067	236	2595	.	.	1.00	9.92	.	.	0	920	.	.
	078*	117	4396	.	.	1.00	23.05	.	.	0	16,338	.	.
	087	89	117	.	.	6.61	5.51	.	.	16	98	.	.
	093	117	4167	.	.	0.00	19.79	.	.	0	12,015	.	.
	104	240	2129	.	.	3.02	7.18	.	.	16	2860	.	.
	117	2063	1141	.	.	12.84	9.09	.	.	402	3602	.	.
	126	243	923	.	.	18.10	35.27	.	.	0	1352	.	.
	139	60	221	.	.	29.03	24.00	.	.	0	1193	.	.
	144	325	6922	.	.	19.87	25.72	.	.	0	4735	.	.
	156	96	1104	.	.	39.68	68.78	.	.	0	2156	.	.
	179	13	3650	.	.	85.22	17.45	.	.	0	3971	.	.
	198	489	2183	.	.	93.67	40.83	.	.	47	1970	.	.
	205	3560	5250	.	.	86.09	64.24	.	.	48	1297	.	.
	218	140	1403	.	.	15.80	6.39	.	.	31	3495	.	.
	228	83	7478	.	.	12.23	6.43	.	.	0	3490	.	.
	239	40	1900	.	.	37.74	32.25	.	.	3	2010	.	.
	274	375	1753	.	.	24.67	0.00	.	.	27	423	.	.
	**Median**	**129**	**2156**	**.**	**.**	**16.95**	**21.42**	**.**	**.**	**0**	**2508**	**.**	**.**
Group 3: 5 doses of 2.70x10^5 PfSPZ<br>CHMI #1 at 20-23 Days<br>CHMI #2 at 170-171 Days	281	75	3096	.	.	27.10	1.09	.	.	2	581	.	.
	306	143	2901	.	.	15.18	17.76	.	.	0	1129	.	.
	310	143	2366	.	.	13.77	24.67	.	.	40	1062	.	.
	325	226	4974	.	.	1.00	27.10	.	.	2	1202	.	.
	335	147	1739	.	.	1.09	15.18	.	.	0	777	.	.
	345	293	1311	.	.	17.76	13.77	.	.	0	828	.	.
	351*	47	12,410	.	12,017	22.42	90.77	.	87.79	0	1726	.	1909
	377	148	936	.	.	7.05	17.57	.	.	1	705	.	.
	383	126	3997	.	.	1.00	38.21	.	.	20	3804	.	.
	397	126	5187	.	.	37.38	23.19	.	.	0	1109	.	.
	401	281	1757	.	.	1.00	11.14	.	.	0	1068	.	.
	411*	40	11,756	.	4877	79.49	105.20	.	92.94	46	7755	.	1788
	420	266	13,862	.	.	8.52	58.84	.	.	30	18,316	.	.
	445*	185	2595	.	1916	96.67	69.67	.	21.12	46	2373	.	460
	453*	1228	3839	.	4357	20.25	76.19	.	54.11	125	10,672	.	656
	469	90	6240	.	.	1.00	41.63	.	.	76	2505	.	.
	473	66	259	.	.	3.65	0.00	.	.	0	124	.	.
	489	10	446	.	.	32.81	0.00	.	.	5	133	.	.
	495	959	3125	.	.	37.81	17.09	.	.	139	763	.	.
	512	85	12,579	.	.	21.83	15.08	.	.	16	8076	.	.
	**Median**	**143**	**3111**	**.**	**4617**	**16.47**	**20.48**	**.**	**70.95**	**4**	**1119**	**.**	**1222**
Group 4: 5 doses of 2.70x10^5 PfSPZ<br>CHMI at 170-171 Days	526	183	11,092	7836	.	1.00	13.00	8.73	.	46	5194	564	.
	538	167	936	615	.	1.00	7.39	7.42	.	0	1821	77	.
	551	126	8701	13,817	.	1.00	13.44	20.87	.	22	4947	1517	.
	563	110	1071	1382	.	1.63	6.34	5.32	.	20	618	124	.
	570	18	681	530	.	1.00	37.78	23.63	.	0	736	99	.
	**Median**	**126**	**1071**	**.**	**.**	**1.00**	**13.00**	**8.73**	**.**	**20**	**1821**	**124**	**.**
Group 2 Controls	054	50	1	.	.	.	.	.	.	.	.	.	.
	160	53	26	.	.	.	.	.	.	.	.	.	.
	240	65	196	.	.	.	.	.	.	.	.	.	.
	253	83	40	.	.	.	.	.	.	.	.	.	.
	**Median**	**-35**	**0.49**	**.**	**.**	**.**	**.**	**.**	**.**	**.**	**.**	**.**	**.**
Group 3 Controls	290	125	109	.	.	.	.	.	.	.	.	.	.
	360	99	53	.	.	.	.	.	.	.	.	.	.
	430	43	1	.	.	.	.	.	.	.	.	.	.
	500	115	81	.	.	.	.	.	.	.	.	.	.
	**Median**	**-39**	**0.62**	**.**	**.**	**.**	**.**	**.**	**.**	**.**	**.**	**.**	**.**
Incomplete Immunization/ Early Dropout	*188*	*49*	*546*	.	.	*11.78*	*5.67*	.	.	*0*	*1087*	.	.
	269	*640*	*1113*	.	.	*46.38*	*0.00*	.	.	*105*	*1249*	.	.
	548	*55*	*1384*	*1270*	.	*1.00*	*10.08*	*13.97*	.	*42*	*325*	*104*	.

For aIFA and ISI, when calculating ratios, all values of 0 were changed to 1. Significant associations were found between PfCSP ELISA and net aIFA (p = 0.0004, R^2 = 0.3636), PfCSP ELISA and ISI (p = 0.012, R^2 = 0.0984), and between net aIFA and ISI (p = 0.012, R^2 = 0.1377). There was only a significant association between PfCSP ELISA and net aIFA (p = 0.0328, R^2 = 0.5014).

*Subjects protected against CHMI.

Table S13. Net and ratio values for PfCSP ELISA, aIFA, and ISI assays. All values in bold were considered to have developed antibodies after immunization, according to the following criteria: In the PfCSP ELISA, samples were considered positive if the difference between the post-immunization OD 1.0 and the pre-immunization OD 1.0 (net OD 1.0) was ≥50 and the ratio of post-immunization OD 1.0 to pre-immunization OD 1.0 (ratio) was ≥3. In the ISI assay, sera with a net ISI activity of ≥10% between post- and pre-immunization sera and a ratio of post- to pre-vaccination ISI activity of ≥3.0 were considered positive. In the aIFA, sera were considered positive for seroconversion if their net AFU $2x10^5$ and AFU $2x10^5$ ratio, calculated respectively by subtracting the pre-vaccination from the post-vaccination AFU $2x10^5$ and dividing the post-vaccination by the pre-vaccination AFU $2x10^5$ were ≥150 and ≥3.0, respectively. For aIFA and ISI, when calculating ratios, all values of 0 were changed to 1.

Group	ID	PfCSP OD 1.0		ISI (80% Inhibition)		aIFA (PfSPZ)	
		Net	Ratio	Net	Ratio	Net	Ratio
**Group 2: 5 doses of $1.35x10^5$ PfSPZ<br><br>CHMI at 19-21 Days**	048	**3130**	**285.55**	**40.52**	**6.53**	**5031**	**5031.00**
	067	**2359**	**11.00**	8.92	9.92	**920**	**920.00**
	078*	**4279**	**37.57**	**22.05**	**23.05**	**16,338**	**16,338.00**
	087	28	1.31	-1.10	0.83	82	6.13
	093	**4050**	**35.62**	**19.79**	**19.79**	**12,015**	**12,015.00**
	104	**1889**	**8.87**	4.16	2.38	**2844**	**178.75**
	117	-922	0.55	-3.75	0.71	**3200**	**8.96**
	126	**680**	**3.80**	17.17	1.95	**1352**	**1352.00**
	139	**161**	**3.68**	-5.03	0.83	**1193**	**1193.00**
	144	**6597**	**21.30**	5.85	1.29	**4735**	**4735.00**
	156	**1008**	**11.50**	29.10	1.73	**2156**	**2156.00**
	179	**3637**	**280.77**	-67.77	0.20	**3971**	**3971.00**
	198	**1694**	**4.46**	-52.84	0.44	**1923**	**41.91**
	205	1690	1.47	-21.85	0.75	**1249**	**27.02**
	218	**1263**	**10.02**	-9.41	0.40	**3464**	**112.74**
	228	**7395**	**90.10**	-5.80	0.53	**3490**	**3490.00**
	239	**1860**	**47.50**	-5.49	0.85	**2007**	**670.00**
	274	**1378**	**4.67**	-24.67	0.00	**396**	**15.67**
	**Median**	**1777**	**10.51**	**-2.43**	**0.84**	**2500**	**1056.50**
**Group 3: 5 doses of $2.70x10^5$ PfSPZ<br><br>CHMI #1 at 20-23 Days<br><br>CHMI #2 at 170-171 Days**	281	**3021**	**41.28**	-26.01	0.04	**579**	**290.50**
	306	**2758**	**20.29**	2.58	1.17	**1129**	**1129.00**
	310	**2223**	**16.55**	10.90	1.79	**1022**	**26.55**
	325	**4748**	**22.01**	**26.10**	**27.10**	**1200**	**601.00**
	335	**1592**	**11.83**	**14.09**	**13.93**	**777**	**777.00**
	345	**1018**	**4.47**	-3.99	0.78	**828**	**828.00**
	351*	**12363**	**264.04**	**68.35**	**4.05**	**1726**	**1726.00**
	377	**788**	**6.32**	10.52	2.49	**704**	**705.00**
	383	**3871**	**31.72**	**37.21**	**38.21**	**3784**	**190.20**
	397	**5061**	**41.17**	-14.19	0.62	**1109**	**1109.00**
	401	**1476**	**6.25**	**10.14**	**11.14**	**1068**	**1068.00**
	411*	**11716**	**293.90**	25.71	1.32	**7709**	**168.59**
	420	**13596**	**52.11**	**50.32**	**6.91**	**18,286**	**610.53**
	445*	**2410**	**14.03**	-27.00	0.72	**2327**	**51.59**
	453*	**2611**	**3.13**	**55.94**	**3.76**	**10,547**	**85.38**
	469	**6150**	**69.33**	**40.63**	**41.63**	**2429**	**32.96**
	473	**193**	**3.92**	-3.65	0.00	124	124.00
	489	**436**	**44.60**	-32.81	0.00	128	26.60
	495	**2166**	**3.26**	-20.72	0.45	**624**	**5.49**
	512	**12494**	**147.99**	-6.75	0.69	**8060**	**504.75**
	**Median**	**2685**	**21.15**	**10.33**	**1.56**	**1119**	**397.63**
**Group 4: 5 doses of $2.70x10^5$ PfSPZ<br><br>CHMI at 170-171 Days**	526	**10909**	**60.61**	**12.00**	**13.00**	**5148**	**112.91**
	538	**769**	**5.60**	6.39	7.39	**1820**	**1821.00**
	551	**8575**	**69.06**	**12.44**	**13.44**	**4925**	**224.86**
	563	**961**	**9.74**	4.71	3.89	**598**	**30.90**
	570	**663**	**37.83**	**36.78**	**37.78**	**735**	**736.00**
	**Median**	**961**	**37.83**	**12**	**13.00**	**1820**	**224.86**
**Group 2 Controls**	054	-49	0.02	.	.	.	.
	160	-27	0.49	.	.	.	.
	240	**131**	**3.02**	.	.	.	.
	253	-43	0.48	.	.	.	.
	**Median**	**-35**	**0.49**	**.**	**.**	**.**	**.**
**Group 3 Controls**	290	-16	0.87	.	.	.	.
	360	-46	0.54	.	.	.	.
	430	-42	0.02	.	.	.	.
	500	-34	0.70	.	.	.	.
	**Median**	**-38**	**0.62**	**.**	**.**	**.**	**.**
**Incomplete Immunization/ Early Dropout**	*188*	***497***	***11.14***	*-6.11*	*0.48*	***1087***	***1087.00***
	269	*473*	*1.74*	*-46.38*	*0.00*	***1144***	***11.90***
	548	***1329***	***25.16***	*9.08*	*10.08*	***283***	***7.74***

*Subjects protected against CHMI

Table S14. Antibodies to well-defined Pf proteins 2 weeks after the 5th dose of PfSPZ Vaccine by ELISA. Proteins include those first expressed in PfSPZ (PfCSP, PfMSP5, PfAMA-1, PfCelTos), early liver stages (PfLSA1, PfEXP1), and late liver stages (PfMSP1, PfEBA175). Antibody levels were the serum dilution at which the optical density was 1.0 (OD 1.0). Net OD 1.0 was the OD 1.0 of post-immunization serum minus the OD 1.0 of pre-immunization serum. A post-immunization serum was considered positive the net OD 1.0 was ≥ 50 (net OD 1.0 ≥ 50) and the ratio of post immunization OD 1.0 to pre-immunization OD 1.0 was ≥ 3.

Protein	Group								
	Group 2 (5 doses of 1.35x10^5 PfSPZ, CHMI at 19-21 days)			Group 3 (5 doses of 2.7x10^5 PfSPZ, CHMI at 20-23 days)			Group 4 (5 doses of 2.7x10^5 PfSPZ, CHMI at 170-171 days)		
	No. Positive /No. Tested (%)	Net OD 1.0 of Positives (Median)	OD 1.0 Ratio of Positives (Median)	No. Positive /No. Tested (%)	Net OD 1.0 of Positives (Median)	OD 1.0 Ratio of Positives (Median)	No. Positive /No. Tested (%)	Net OD 1.0 of Positives (GM)	OD 1.0 Ratio of Positives (GM)
**1st Expressed in Sporozoites**									
PfCSP	15/18 (83%)	1889	11.50	20/20 (100%)	2685	21.15	5/5 (100%)	961	37.83
PfCelTOS	1/18 (6%)	56*	3.07*	1/20 (5%)	75*	75.00*	0/5		
PfMSP5	2/18 (11%)	257	3.86	3/20 (15%)	182	5.51	0/5		
PfAMA1	4/18 (22%)	1891	4.97	9/20 (45%)	486	5.13	1/5 (20%)	236*	3.46*
**1st Expressed in Early Liver Stages**									
PfEXP1	0/18			0/20			0/5		
PfLSA1	0/18			1/20 (5%)	853*	6.72*	0/5		
**1st Expressed in Late Liver Stages**									
PfMSP1	1/18 (6%)	242*	243.00*	4/20 (20%)	109	5.38	0/5		
PfEBA175	1/18 (6%)	109*	110.00*	1/20 (5%)	103,195*	2458.02*	0/5		

* For these values, the single value is recorded, not the median.

Figure S1

A

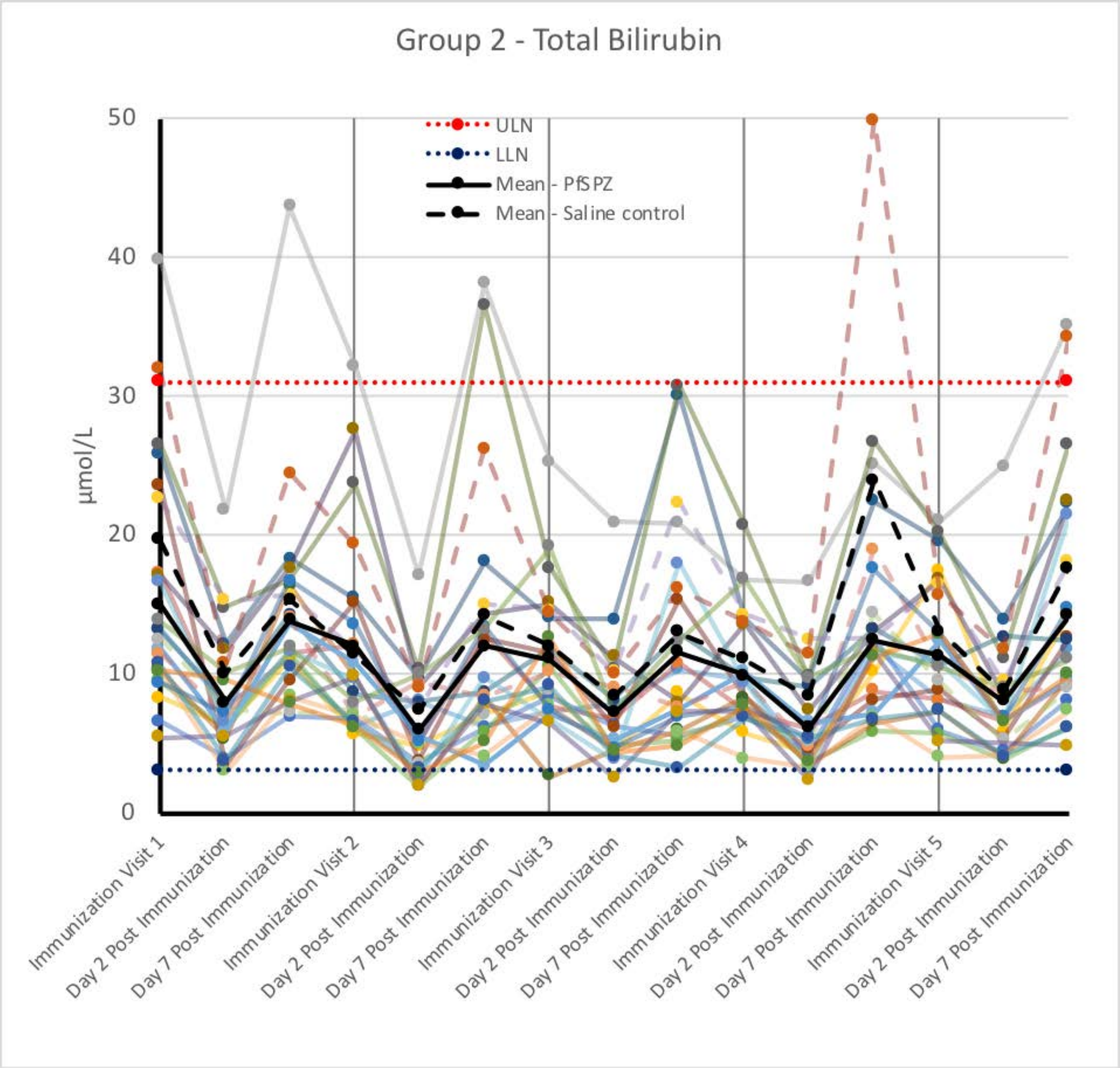

Group 2 - Total Bilirubin
ULN
LLN
Mean - PfSPZ
Mean - Saline control
µmol/L
50
40
30
20
10
0
Immunization Visit 1
Day 2 Post Immunization
Day 7 Post Immunization
Immunization Visit 2
Day 2 Post Immunization
Day 7 Post Immunization
Immunization Visit 3
Day 2 Post Immunization
Day 7 Post Immunization
Immunization Visit 4
Day 2 Post Immunization
Day 7 Post Immunization
Immunization Visit 5
Day 2 Post Immunization
Day 7 Post Immunization

B

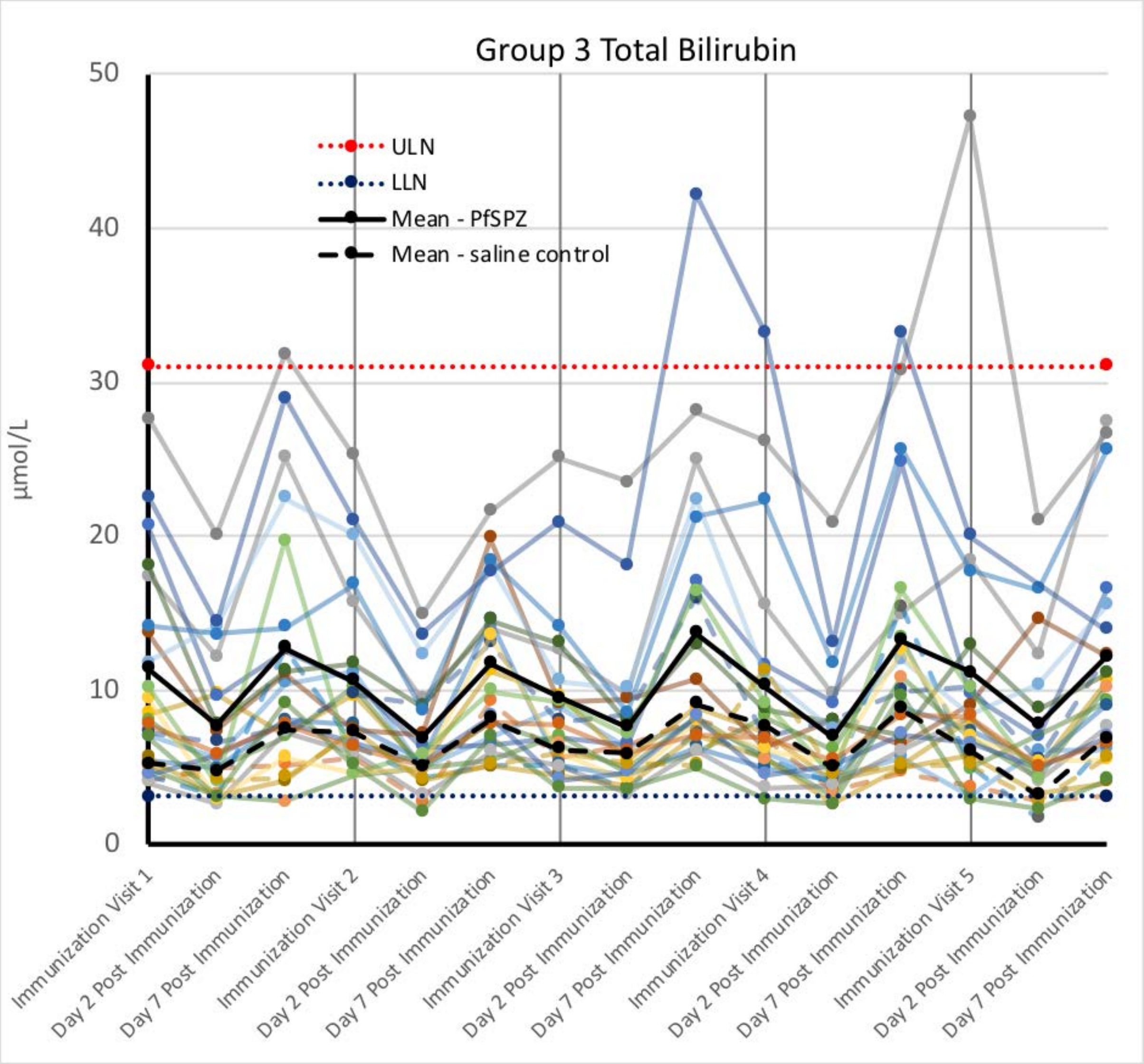

C

	Difference in Total Bilirubin (µmol/L) Between Day 0 and Day 2			
	Group 2 (G2)		Group 3 (G3)	
	Vaccine	Control	Vaccine	Control
Mean	4.36	3.61	2.72	2.00
Standard Deviation	4.00	2.74	4.64	2.09
Median	3.9	4.1	2.25	1.9

Range	(-3.9, 18.5)	(-2.3, 10.2)	(-12.4, 26.2)	(-1.1, 6.8)
95% CI	(3.45, 5.27)	(2.16, 5.07)	(1.67, 3.77)	(0.89, 3.11)

Figure S1. Trends in total bilirubin, Group 2 (panel A) and Group 3 (panel B) volunteers. Trend lines are presented for each volunteer and as mean trend lines for PfSPZ Vaccine and saline control subjects. Nadirs in total bilirubin in Group 2 and 3 volunteers occurred consistently 2 days after each DVI and returned to baseline by day 7. Mean decline in total bilirubin from day of immunization to 2 days after was 3.41 µmol/L and was statistically significant ($p < 0.0001$, 95% CI 2.81-4.01). Significant declines occurred in all 4 groups after each immunization (panel C). Intragroup comparisons (Group 2 vaccinees vs. Group 2 controls, Group 3 vaccinees vs. Group 3 controls, Group 2 vaccinees vs. Group 3 vaccinees and Group 2 controls vs. Group 3 controls) were not statistically significant ($p > 0.05$ for all comparisons). Changes in diet, particularly in situations of refeeding after caloric deprivation, have been associated with transient decreases in total bilirubin.[36] Since all volunteers, all of whom were students living in Dar es Salaam, were housed and fed at the research facility for 2 days after each immunization, it appears this is the most likely explanation for this finding.
